# Integrin-αvβ3 is a Therapeutically Targetable Fundamental Factor in Medulloblastoma Tumorigenicity and Radioresistance

**DOI:** 10.1158/2767-9764.CRC-23-0298

**Published:** 2023-12-07

**Authors:** William Echavidre, Jérôme Durivault, Célia Gotorbe, Thays Blanchard, Marina Pagnuzzi, Valérie Vial, Florian Raes, Alexis Broisat, Rémy Villeneuve, Régis Amblard, Nicolas Garnier, Cécile Ortholan, Marc Faraggi, Benjamin Serrano, Vincent Picco, Christopher Montemagno

**Affiliations:** 1 Département de Biologie Médicale, Centre Scientifique de Monaco, Monaco, Monaco.; 2 Université de Grenoble Alpes, INSERM, LRB, Grenoble, France.; 3 Medical Physics Department, Centre Hospitalier Princesse Grace, Monaco, Monaco.; 4 Radiotherapy Department, Centre Hospitalier Princesse Grace, Monaco, Monaco.; 5 Nuclear Medicine Department, Centre Hospitalier Princesse Grace, Monaco, Monaco.

## Abstract

Medulloblastoma is one of the most prevalent solid tumors found in children, occurring in the brain's posterior fossa. The standard treatment protocol involves maximal resection surgery followed by craniospinal irradiation and chemotherapy. Despite a long-term survival rate of 70%, wide disparities among patients have been observed. The identification of pertinent targets for both initial and recurrent medulloblastoma cases is imperative. Both primary and recurrent medulloblastoma are marked by their aggressive infiltration into surrounding brain tissue, robust angiogenesis, and resistance to radiotherapy. While the significant role of integrin-αvβ3 in driving these characteristics has been extensively documented in glioblastoma, its impact in the context of medulloblastoma remains largely unexplored. Integrin-αvβ3 was found to be expressed in a subset of patients with medulloblastoma. We investigated the role of integrin-αvβ3 using medulloblastoma-derived cell lines with β3-subunit depletion or overexpression both *in vitro* and *in vivo* settings. By generating radioresistant medulloblastoma cell lines, we uncovered an increased integrin-αvβ3 expression, which correlated with increased susceptibility to pharmacologic integrin-αvβ3 inhibition with cilengitide, a competitive ligand mimetic. Finally, we conducted single-photon emission computed tomography (SPECT)/MRI studies on orthotopic models using a radiolabeled integrin-αvβ3 ligand (^99m^Tc-RAFT-RGD). This innovative approach presents the potential for a novel predictive imaging technique in the realm of medulloblastoma. Altogether, our findings lay the foundation for employing SPECT/MRI to identify a specific subset of patients with medulloblastoma eligible for integrin-αvβ3–directed therapies. This breakthrough offers a pathway toward more targeted and effective interventions in the treatment of medulloblastoma.

**Significance:**

This study demonstrates integrin-αvβ3’s fundamental role in medulloblastoma tumorigenicity and radioresistance and the effect of its expression on cilengitide functional activity.

## Introduction

Brain tumors represent the second most common childhood tumors, following hematologic cancers (1). Medulloblastoma is among the most common brain tumor in the pediatric population, accounting for approximately 20% of all childhood brain tumors (2). Medulloblastomas typically originate in the posterior fossa, primarily within the cerebellum, often manifesting early in life (3). The current standard of care for medulloblastomas includes a maximal tumor resection followed by craniospinal irradiation and chemotherapy (4). Recent advances in the molecular understanding of medulloblastoma pathophysiology have delineated four distinct prognosis molecular subgroups: wingless (WNT), sonic hedgehog (SHH), group 3, and group 4. Nevertheless, the clinical translation of these observations is just starting to emerge. Therefore, the treatment strategies employed for medulloblastomas are currently hinged upon categorizing patients into standard- or high-risk groups according to the presence of metastases, age, and extent of postoperative residual disease.

Standard-risk patients (constituting ∼70% of medulloblastomas) are typically children ages over 3 years with no evidence of disseminated disease and postoperative residual tumor of <1.5 cm^2^. In such instances, the 5-year postdiagnosis overall survival (OS) stands as 85%. If at least one of these criteria is unmet, the patient is considered high-risk, and the 5-year OS drops to 60% (5). Despite the recent identification of four core molecular subgroups (SHH, WNT, group 3, and group 4), clinical stratification of the patients still mainly relies on the aforementioned clinical features. In addition, the tumors of about 30% of the patients originally included in the standard-risk group eventually relapse. Consequently, the accurate identification of high-risk medulloblastomas and the effective management of recurrent medulloblastomas remain substantial challenges. These challenges are further accentuated by the absence of robust prognostic markers and limited therapeutic avenues for cases of recurrence. These issues highlight the need to refine medulloblastoma diagnostic criteria with new prognostic and predictive markers and therapeutic options designed explicitly for recurring disease.

Over the past decades, our understanding of the molecular pathogenesis of malignant brain tumors such as gliomas has yielded insights into novel regulators of key processes in glioblastoma (GBM) cell growth, invasion, and angiogenesis (6). Among these factors, integrins have emerged as pivotal players and promising therapeutic targets for GBM. Integrins are a large family comprising noncovalent heterodimeric complexes involving 18 α and 8 β subunits, possibly forming at least 24 different receptor types (7). Integrins are membrane-bound receptors specific to interactions between cells and extracellular matrix (ECM) components such as fibronectin, laminin, and collagen. Upon binding to their ligands, integrins activate downstream signaling pathways that regulate many cellular effects under physiologic and pathologic conditions. While integrin-encoding genes are rarely mutated in cancer, dysregulation of their expression level or integrin signaling is common, especially in brain tumors (8).

Among these integrin complexes, integrin-αvβ3 was the first to be found aberrantly expressed in high-grade brain tumors. Integrin-αvβ3 belongs to the integrin subtypes that recognize the tripeptide Arg-Gly-Asp (RGD) sequence found in many ECM proteins, including fibronectin and vitronectin. Many studies have highlighted the central role of αvβ3 in sustaining the proliferation and invasive properties of brain tumors through the activation of focal adhesion kinase (FAK), leading to the activation of downstream pathways, including the PI3K/protein kinase B (Akt) and ERK/MAPK pathways (6). In addition to these features, αvβ3 was found necessarily and sufficiently to reprogram tumor cells toward a cancer stem cells phenotype (tumor initiation, self-renewal) in several solid tumors (9). These significant insights have been bolstered by clinical investigations, where the expression of αvβ3 has been linked to unfavorable prognosis and reduced time-to-progression (10).

Given their involvement in tumor progression, integrins have garnered significant attention as attractive therapeutic targets for solid tumors. Therefore, several strategies have been explored to target integrin-αvβ3, including the use of small molecules. They include a cyclic pentapeptide blocking the RGD binding site (cilengitide; cyclo-Arg-Gly-Asp-DPhe-NMe-Val) that acts as a selective αvβ3 inhibitor. Cilengitide was found to impair GBM tumorigenesis by hampering angiogenesis, cell proliferation, and invasion. This agent was subsequently evaluated in clinical studies (11, 12). Cilengitide had moderate antitumor efficacy in patients with GBM, with antitumor effects restricted to high-αvβ3–expressing tumors, highlighting the potential need to stratify patients based on αvβ3 expression in their tumors (13). In this context, the advent of nuclear medicine is currently one of the greatest components of theranostics concepts. As a molecular probe, radiolabeled RGD was developed for αvβ3-based imaging and therapy of solid tumors, including GBM (8). Nevertheless, such studies remain to be conducted for medulloblastoma.

In this novel study, we aimed to evaluate the role and the relevance of integrin-αvβ3 in medulloblastoma. First, the integrin-αvβ3 expression was determined in patient-derived samples and naïve and radioresistant medulloblastoma cell lines. Then, integrin-αvβ3’s role was explored using genetic depletion of the β3-subunit or cilengitide *in vitro* and *in vivo* in orthotopic medulloblastoma mouse models. Finally, molecular imaging with ^99m^Tc-radiolabeled-RGD peptides was used to noninvasively measure integrin-αvβ3 expression in medulloblastomas. By integrating these diverse methodologies, our study aimed to provide a comprehensive understanding of the role and relevance of integrin-αvβ3 in the complex landscape of medulloblastoma.

## Materials and Methods

### Cell Lines and Culture Conditions

Human medulloblastoma cell lines were used in this study. DAOY (ATCC: HTB-186; SHH group) and CHLA-01-Med (ATCC: CRL-3021) were purchased from the ATCC, HD-MB03 [Deutsche Sammlung von Mikroorganismen und Zellkulturen (DMSZ): ACC 740; group 3] from DMSZ. D458 (Cellosaurus: CVCL_1161; group 3) was from Dr. Celio Pouponnot (Institut Curie, Paris, France). ONS-76 (Cellosaurus: CVCL_1624, SHH group) was obtained from Dr. Gilles Pagès [Institute for Research on Cancer and Aging-Nice, Nice, France, originally obtained from Health Science Research Resources Bank (IFO50355)]. All cell lines were tested negative for *Mycoplasma* during routine surveillance [PlasmoTest—Mycoplasma Detection Kit (Mycostrip, Invivogen)] and reauthentication was not performed. DAOY, D458, and ONS-76 cells were cultured in DMEM supplemented with 1 mmol/L sodium pyruvate, 2 mmol/L Glutamax, and 7.5% FBS. CHLA-01-Med cells were cultured in DMEM/F12 supplemented with 2% B-27, 20 ng/mL EGF, and 20 ng/mL basic FGF. HD-MB03 cells were cultured in RPMI medium supplemented with 7.5% FBS. All cell lines were cultured less than 10 passages from thawed vials and maintained at 37°C in 5% CO_2_ humidified incubator, and mostly subcultured twice a week.

### Genetic Disruption of β3-integrin Using CRISPR-Cas9, β3-integrin Expression Rescue, and Lentiviral Transductions

DAOY wildtype (WT) cells were transfected using jetPEI (Polyplus, 101000053) according to the manufacturer's instructions with pSpCas9(BB)-2A-GFP (PX458) plasmid (a gift from Feng Zhang; Addgene plasmid #48138; http://n2t.net/addgene:48138; research resource identifier: Addgene_48138) containing clustered regularly interspaced short palindromic repeats (CRISPR)-CRISPR-associated protein 9 (Cas9) targeting regions for the first [guide RNA (gRNA): 5′-GAGGCGGACGAGATGCGAGCG-3′] and 10th (gRNA 5′-AGACGGGCTGACCCTCCCGG-3′) exons of the β3-integrin gene (*ITGB3*). The *ITGB3* sequence (pcDNA3.1-beta-3 was a gift from Timothy Springer; Addgene plasmid # 27289) was subcloned into pLenti6.3/TO/V5-Blasti (A11144, Thermo Fisher Scientific) to create β3-integrin expression plasmids. Lentiviruses were produced by triple transfection of HEK-293T cells with the lentiviral transfer vector pLenti6.3/TO/V5-Blasti and the packaging plasmids psPAX2 (Addgene plasmid #12260) and pMD2.G (Addgene plasmid #12259), a kind gift from Didier Trono, at a ratio of 0.3:0.3:0.1. Transfection was performed using jetPEI (101000053, Polyplus). The viral supernatant was collected 48 hours after transfection, filtered through a 0.45 µm filter, and added to the target cells.

For *in vivo* tumorigenesis assays, DAOY-derived and HD-MB03–derived cells were transduced with lentiviral particles containing RFP-Luc (Lenti-One RFP-Luc, GEG Tech) at a multiplicity of infection of 0.3 in the presence of hexadimethrine bromide (4 µg/mL).

### qRT-PCR

Real-time qRT-PCR analyses were performed using human cerebellar mRNA (Biochain) and medulloblastoma cell lines. mRNAs were prepared using a Nucleospin RNA Kit (Macherey-Nagel), and cDNA synthesis was performed using a Maxima First Strand cDNA Synthesis Kit for qRT-PCR with dsDNase (Thermo Fisher Scientific). Quantitative PCR analyses were performed on an Applied Biosystems StepOnePlus System using TB Green Premix Ex Ta (Tli RNase H Plus; Takara Bio) reagents. The primers used are listed in Supplementary Table S1. Relative expression levels were determined using the ΔCt method and normalized to the reference gene 36B4. Results are expressed relative to the normal cerebellum.

### Immunoblotting

Cells were lysed in 1.5 × Laemmli buffer, and protein concentrations were determined using the Pierce bicinchoninic acid Protein Assay (Thermo Fisher Scientific). Protein extracts (40 µg) were separated via electrophoresis on 10% SDS-polyacrylamide gels and transferred to polyvinylidene difluoride membranes (Millipore). Membranes were blocked in 2% milk PBS and incubated with the following anti-human antibodies: rabbit β3-integrin [1:1,000; 13166, Cell Signaling Technology (CST)], rabbit p-FAK (1:1,000; 8566, CST), rabbit FAK (1:1,000; 71433, CST), mouse p-Akt (1:1,000; 4051, CST), rabbit Akt (1:1,000; 9272, CST), rabbit p-ERK1/2 (1:1,000; 4370, CST), rabbit ERK1/2 (1:1,000; 4695, CST), and rabbit PARP (1:1,000, 9542; CST). Actin was used as the protein loading control (1:5,000; MA5-15739, Thermo Fisher Scientific). Immunoreactive bands were detected with horseradish peroxidase-coupled anti-mouse or anti-rabbit antibodies (CST) using the ECL System (Merck Millipore; WBKLS0500). Immunoblot analysis was performed using the LI-COR Odyssey Imaging System.

### Measurement of Integrin-αVβ3 Expression by FACS

DAOY-derived and HD-MB03–derived cells were seeded into 6-well dishes for 24 hours. Cells were rinsed with PBS, detached with accutase (00-4555-56, Thermo Fisher Scientific), and collected. Cells (250,000) were incubated with a mouse anti-αvβ3 antibodies (1 µg/mL; ab190147, Abcam) for 1 hour in PBS/1% BSA on ice. Next, cells were centrifuged and washed twice with PBS. Then, cells were incubated with a goat anti-mouse IgG secondary antibody coupled to AlexaFluor488 (1 µg/mL; A-11029, Invitrogen). All experiments were performed in triplicate. Ten thousand events were analyzed per sample using a BD FACS Melody cytometer (BD Biosciences). Data were analyzed using the FlowJo software.

### Cell Adhesion Assays

Cell adhesion to ECM proteins was performed using a CytoSelect 48-well Cell Adhesion Assay Kit (Cell Biolabs) according to the manufacturer's protocol. Briefly, 100,000 DAOY-derived and 250,000 HD-MB03–derived cells were collected in 200 µL of serum-free medium and added to the prewarmed ECM adhesion plate. The plates were incubated at 37°C for 90 minutes in a CO_2_ incubator. The wells were washed three times with PBS and stained with 200 µL of cell staining solution for 10 minutes at room temperature. After washing and drying, the stained cells were extracted with extraction solution and incubated in an orbital shaker for 10 minutes. Then, 150 µL of each sample was transferred to a 96-well plate and quantified by measuring absorbance at 560 nm.

To determine cilengitide's IC_50_, 1 µg of fibronectin was plated onto 96-well plates for 60 minutes at 37°C in a CO_2_ incubator. Serum-starved cells were added to the fibronectin-coated wells (10,000 cells for DAOY and 30,000 for HD-MB03) in the presence of serial cilengitide dilutions (0–200 µmol/L), and the plates were incubated for 90 minutes at 37°C with 5% CO_2_. Then, cells were washed three times with PBS and stained for 20 minutes with a 1% Crystal Violet solution at room temperature. After washing with PBS, adherent cells were solubilized with DMSO and quantified by measuring absorbance at 590 nm. The IC_50_ was calculated using the nonlinear regression method in the Prism 8 software (Graphpad Software Inc.).

### Proliferation Assay

Cells (25,000 and 50,000 DAOY-derived and HD-MB03–derived cells, respectively) were seeded onto 6-well plates in triplicate per cell line and per condition. Cell proliferation was assessed by daily trypsinization and counting (Coulter Z1; Beckman) for 4 days (96 hours). Both adherent and floating cells were counted. The cell proliferation index was calculated as the percentage of day 0 by standardizing each measurement to the cell number obtained 24 hours after seeding (day 0) or after initiation of cilengitide (0.5 or 2.5 µmol/L) or LM609 (1 or 10 µg/mL) treatment.

### MTT Assay

Cells (3,000 and 10,000 for DAOY and HD-MB03 cells, respectively) were seeded in 96-well plates (Corning Inc.) in 100 µL of medium per well. Cilengitide concentrations from 0 to 500 µmol/L were tested. Its effect was determined using the 3-(4,5-dimethylthiazol-2yl)-diphenyltetrazolium bromide (MTT) colorimetric assay (Sigma-Aldrich) according to the manufacturer's instructions. When applicable, the EC_50_ was calculated using the nonlinear regression method in the Prism 5 software (Graphpad Software Inc.).

### Migration and Invasion Assays

*In vitro* migration and invasion assays were performed in Boyden chambers (Corning) with porous membranes coated or not with 50 µg/mL Matrigel for 2 hours at 37°C to assess invasion or migration, respectively. DAOY (25,000 cells) and HD-MB03 (75,000 cells) cells were placed in serum-free DMEM in the upper compartment [with or without cilengitide (0.5 or 2.5 µmol/L) or LM609 (1 or 10 µg/mL) treatment] and DMEM in the lower compartment of the chambers. After 12 and 24 hours incubation for DAOY and HD-MB03, respectively, cells were stained with Giemsa for 30 minutes. Invasive cells were counted under the microscope. Results are expressed as % of the control.

### Cell Death

Cells were seeded in 12-well plates (50,000–150,000 cells per well, triplicate per condition) at 37°C/5% CO_2_ in their respective media. Cells were treated with cilengitide (20 µmol/L) for 48 hours. Both floating and adherent cells were collected and centrifuged. Then, cell pellets were resuspended in FACS buffer (PBS, 0.2% BSA, and 2 mmol/L ethylenediaminetetraacetic acid) and stained with 2 µg/mL propidium iodide (PI; Invitrogen). PI was added just before the analysis. Experiments were performed at least three times, 10,000 events were analyzed per sample using a BD FACSMelody cytometer, and data were analyzed using the FlowJo software.

### Generation of Radioresistant Cells

Adherent cells were irradiated with 8 Gy of X-rays using a 6 MeV Novalis TrueBeam linear accelerator (Novalis TrueBeam STX, 3 Gy/minute) and returned to the incubator afterward. Radioresistant cell lines (DAOY-RR and HD-MB03-RR) were developed from their respective parental cell lines (DAOY and HD-MB03) by weekly exposure to single fractions of radiation. An initial dose of 2 Gy was followed by incremental doses for 12 weeks (from 2 to 8 Gy). During this period, cells received a total of 60 Gy.

### Immunofluorescence

Tumor sections (5 µm cryostat sections) were fixed in 4% paraformaldehyde for 10 minutes at room temperature and blocked in 1% horse serum in TBS for 1 hour. The sections were incubated with rat monoclonal anti-mouse platelet and endothelial cell adhesion molecule 1 (PECAM1/CD31; clone MEC 13.3, 1:1,000; BD Pharmingen) and monoclonal anti-mouse α-smooth muscle actin (αSMA; A2547, 1:1,000; Sigma) or anti-Ki67 (ab16667, 1:500; Abcam) antibodies diluted in TBS containing 1% BSA at 1:100 overnight at room temperature, and thereafter washed with TBS containing 0.025% Triton. Afterward, the preparations were incubated with anti-rabbit Alexa488- (#4412, CST) and anti-mouse Alexa-555–coupled secondary antibodies. Next, preparations were washed with TBS containing 0.025% Triton, and the nuclei were counterstained with Hoechst33342 (Thermo Fisher Scientific). Fluorescence images of the cells were taken with a DMI400 (Leica Microsystems) inverted microscope equipped with a 10 × objective (Leica Microsystems) and a Zyla 5.5 camera (Andor Technologies). Preparations were mounted and imaged using a Leica microscope (DMI4000B, Leica) and counted at 10 × (CD31/αSMA) or 40 × (Ki67) magnification with Fiji software (14).

### Biomax Tissue Array

Medulloblastoma tissue microarrays (catalog number BC17012c) were purchased from US Biomax. Integrin-αvβ3 expression was assessed by IHC. Briefly, after deparaffinization, sections were saturated with TBS containing 1% BSA and 1% horse serum for 1 hour at room temperature. Tumor sections were incubated with a mouse anti-human anti-integrin-αvβ3 antibody (1:100; ab7166, Abcam) overnight at 4°C. Then, sections were washed with TBS containing 0.25% Triton and incubated with a secondary biotinylated antibody (Vectastain ABC-HRP Kit, peroxidase, PK-4002, vector), followed by 3,3′-diaminobenzidine stain (Vector).

### Intracranial Orthotopic Tumor Xenograft Models

DAOY-Luc spheroids (eight per animal) or HD-MB03-Luc cell suspensions (5,000 cells per animal) were stereotaxically implanted into the brains of 9-week-old Rj:NMRI-Foxn1 nude (nu/nu) female mice (Janvier Labs). Briefly, DAOY spheroids were generated with 2,500 cells grown for 48 hours in ultralow-adhesion spheroid 96-well plates (Corning). DAOY-Luc spheroids and HDMB-03-Luc cells were implanted into the left cerebellar hemisphere (2 mm posterior, 1.5 mm left of the lambda point, and 2.5 mm deep) using a Hamilton syringe fitted with a needle (Hamilton) and following a previously described procedure (15). Cilengitide was resuspended in 200 µL of an aqueous solution of 5% DMSO in PBS. Mice were administered 300 µg cilengitide three times per week via intraperitoneal injection. Intraperitoneal with vehicle solution served as a control. Survival of mice was evaluated by daily monitoring using neuropathological symptoms, including gait abnormalities and weight loss >10% as endpoints. At least 6 mice per group were selected to achieve sufficient statistical power.

### Bioluminescence Imaging

Mice were imaged 2 days after cell implantation, and tumor growth was evaluated by bioluminescent imaging (BLI; IVIS, PerkinElmer). Mice were monitored for up to 170 days (DAOY-derived cells) or 50 days (HD-MB03–derived cells) after intraperitoneal injection of 3.3 mg of d-Luciferin dissolved in 100 µL of PBS. Ten minutes postinjection, mice were anesthetized with isoflurane and imaged with an IVIS Spectrum (field of view: C; binning: medium, f-stop: 1; exposure time: 1 minute). Bioluminescence signals [total flux (photons/second)] were quantified using Living Image 2.0 (Caliper Life Sciences).

### Single-photon Emission Computed Tomography/MRI and Autoradiography

The *in vivo* imaging studies used 12 nine-week-old Rj:NMRI-Foxn1 nude (nu/nu) female mice (Janvier Labs). Acquisitions were made using dedicated MRI and single-photon emission computed tomography (SPECT)/CT MRI systems on 6 mice bearing DAOY tumors and 6 mice bearing HD-MB03 tumors 7 and 4 weeks after tumor implantation, respectively, (Nanoscan PET/MRI3T and Nanoscan SPECT/CT; Mediso). Brain tumors were visualized using T1-weighted coronal images acquired after shimming and 2 minutes after intravenous administration of 100 µL of DOTAREM (0.5 mmol/mL gadoteric acid) using a fast spin-echo MRI sequence (repetition/echo time = 526/27.8 ms, 12 average, 32 slices, and voxel size = 0.34 × 0.34 × 1 mm^3^). Once tumors were observable on MRI acquisitions, SPECT/CT acquisitions were performed 1 hour after intravenous injection of 63.0 ± 12.6 MBq of ^99m^Tc-RAFT-RGD. SPECT quantification was performed using volumes of interest located within the tumor and contralateral healthy cerebellum, and the tumor-to-contralateral ratio was determined (VivoQuant, Invicro). Then, the animals were euthanized using CO_2_ inhalation, and the brain was harvested. Following *ex vivo* gamma-well counting, left and right cerebellum samples were frozen, and 20-µm-thick slices were obtained (NX50V, MM France). After overnight exposure to a phosphor-screen (BAS-IP SR 2025 E, Cytiva), autoradiographic images were acquired (Typhoon IP, Cytiva) and quantified using dedicated software (ImageQuantTL). Regions of interest were drawn around tumor lesions and healthy contralateral cerebellum on a minimum of three slices. Results were background corrected and expressed as average tumor-to-contralateral ratios.

### Study Approval

All animal experiments were conducted in strict accordance with the recommendations of the Guide for the Care and Use of Laboratory Animals. All animal studies were approved in advance by the local animal care committee (Veterinary Service and Direction of Sanitary and Social Action of Monaco; APAFIS # 19480-2019022616164184v4).

### Statistical Analysis

Data are presented as mean ± SEM. Data were compared between groups using nonparametric Mann–Whitney (two groups) or two-way ANOVA corrected for multiple comparisons using Sidak test (>2 groups). Tumor growth was compared between groups using two-way ANOVA corrected for multiple comparisons using Sidak test. Survival was compared between groups using the log-rank test (Mantel–Cox). A *P* < 0.05 was statistically significant.

### Data Availability Statement

The data generated in this study are available upon request from the corresponding author.

## Results

### Integrin-αvβ3 Expression in Medulloblastomas

**FIGURE 1:**
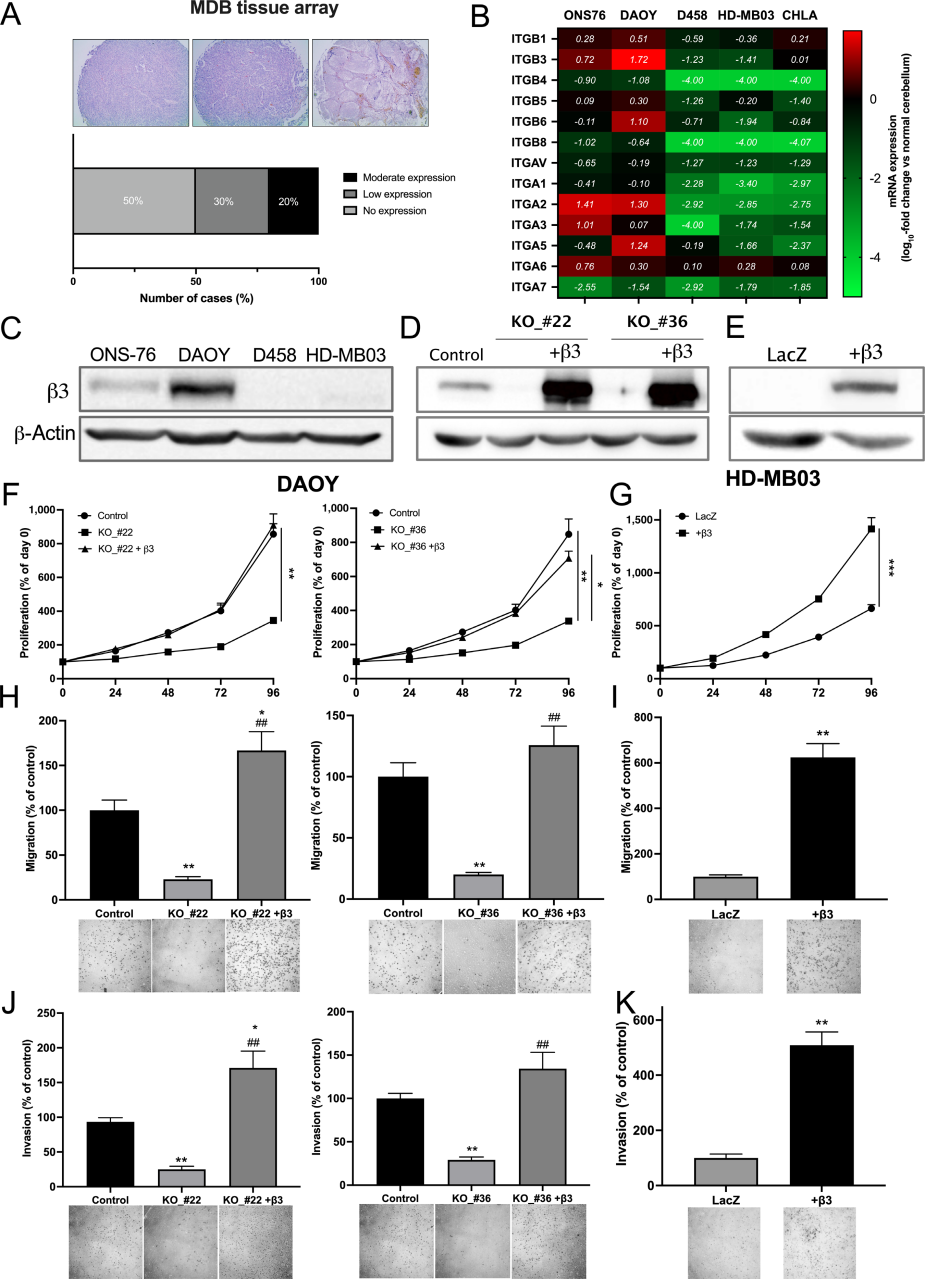
Integrin-αvβ3 is differentially expressed in medulloblastoma cell lines and promotes their proliferation, migration, and invasion. **A,** IHC staining of integrin-αvβ3 in human medulloblastomas using tissue array slides (US Biomax, *n* = 20). Staining intensity was classified as no, low, or moderate expression. **B,** A heat map showing integrin gene expression in medulloblastoma cell lines quantified by qRT-PCR. Results are expressed in comparison to normal cerebellum (log_10_-fold change). The mean values of three independent experiments are shown. **C,** A Western blot analysis of β3-integrin protein expression in medulloblastoma cell lines. **D** and **E,** Representative Western blots of β3-integrin expression in DAOY (control vs. KO and β3-integrin–overexpressing cells) and HD-MB03 (LacZ vs. β3-integrin–overexpressing cells) cell lines. Three independent experiments were performed. The proliferation of DAOY_Control, KO_#22, KO_#22-rescue (**F,** left), KO_#36, KO_#36-rescue (**F,** right), and HD-MB03-LacZ and overexpressing-β3-integrin (**G**). Proliferation rates represent the percentage change versus day 0. Three independent experiments were performed, and data are presented as mean ± SEM. The migration of DAOY_Control, KO_#22, KO_#22-rescue (**H,** left), KO_#36, KO_#36-rescue (**H,** right), and HD-MB03-LacZ and overexpressing-β3-integrin (**I**). Serum-starved cells were allowed to migrate for 12 (DAOY) or 24 hours (HD-MB03). Migrations were performed using Boyden chamber assays, and the results are presented as the percentage of control cells. Three independent experiments were performed, and data are expressed as mean ± SEM. The invasion of DAOY_Control, KO_#22, KO_#22-rescue (**J,** left), KO_#36, KO_#36-rescue (**J,** right), and HD-MB03-LacZ and overexpressing-β3-integrin (**K**). Serum-starved cells were allowed to invade for 12 (DAOY) or 24 hours (HD-MB03). Invasions were performed in a Boyden chamber coated with Matrigel, and results are presented as the percentage of control cells. Three independent experiments were performed, and data are presented as mean ± SEM. Key: *, *P* < 0.05; **, *P* < 0.01; ***, *P* < 0.001 versus control; ^##^, *P* < 0.01 versus KO_cells.

Integrin-αvβ3 expression was first assessed in a tissue array of medulloblastoma-derived samples (Fig. 1A). Among the 20 patients analyzed, 20% (4/20) expressed significant integrin-αvβ3 levels. Because several integrins are upregulated in brain tumors, their expression was examined in several medulloblastoma cell lines (ONS-76, DAOY, D458, CHLA-01Med, and HD-MB03). Most integrin genes were overexpressed in DAOY and ONS-76 (SHH group) cell lines but showed low expression in HD-MB03, D458, and CHLA-01 (groups 3 and 4) cell lines (Fig. 1B). Among them, high *ITGB3* expression was detected in DAOY and ONS-76. This observation was confirmed at the protein level with higher expression in DAOY cells (Fig. 1C). To investigate the role of αvβ3 in medulloblastoma tumorigenesis, *ITGB3* was knocked out (KO) in DAOY cells using CRISPR/Cas9 technology, and β3 was overexpressed in DAOY-KO and HD-MB03 cells (Fig. 1D and E; Supplementary Fig. S1).

### Integrin-αvβ3 Promotes Medulloblastoma Cell Proliferation, Migration, and Invasion

Because a major function of integrin-αvβ3 is cell adhesion to the ECM, an adhesion assay was first performed on various ECM proteins (Supplementary Fig. S2). Genetic ablation of the β3-subunit in both KO clones #22 and #36 resulted in significantly decreased adhesion of DAOY cells to fibronectin and fibrinogen. Overexpression of the β3-subunit in DAOY-KO clones or HD-MB03 cells restored cell adhesion to these ECM proteins (Supplementary Fig. S2). The proliferation of DAOY-KO cells was significantly slower than control and β3-overexpressing cells (Fig. 1F). The same results were observed with HD-MB03 cells (Fig. 1G). Analysis of downstream-integrin-αvβ3 signaling revealed decreased pFAK, pAkt, and pERK1/2 levels in DAOY-KO and HD-MB03_WT cells (Supplementary Fig. S3). As a key player in cell adhesion, the role of integrin-αvβ3 in migration and invasion processes was further investigated. Genetic ablation of the β3-subunit decreased cell migration of both KO clones by 80% (Fig. 1H), while its overexpression in HD-MB03 cells increased cell migration by 600% (Fig. 1I). Similar observations were made in invasion assays (Fig. 1J and K). Restitution of β3-integrin in DAOY-KO cells completely abolished the anti-migratory and anti-invasive effects of β3-depletion. Therefore, integrin-αvβ3 promotes medulloblastoma cell proliferation, migration, and invasion.

### Integrin-αvβ3 Promotes Tumorigenesis of Orthotopic Medulloblastoma Xenografts

**FIGURE 2:**
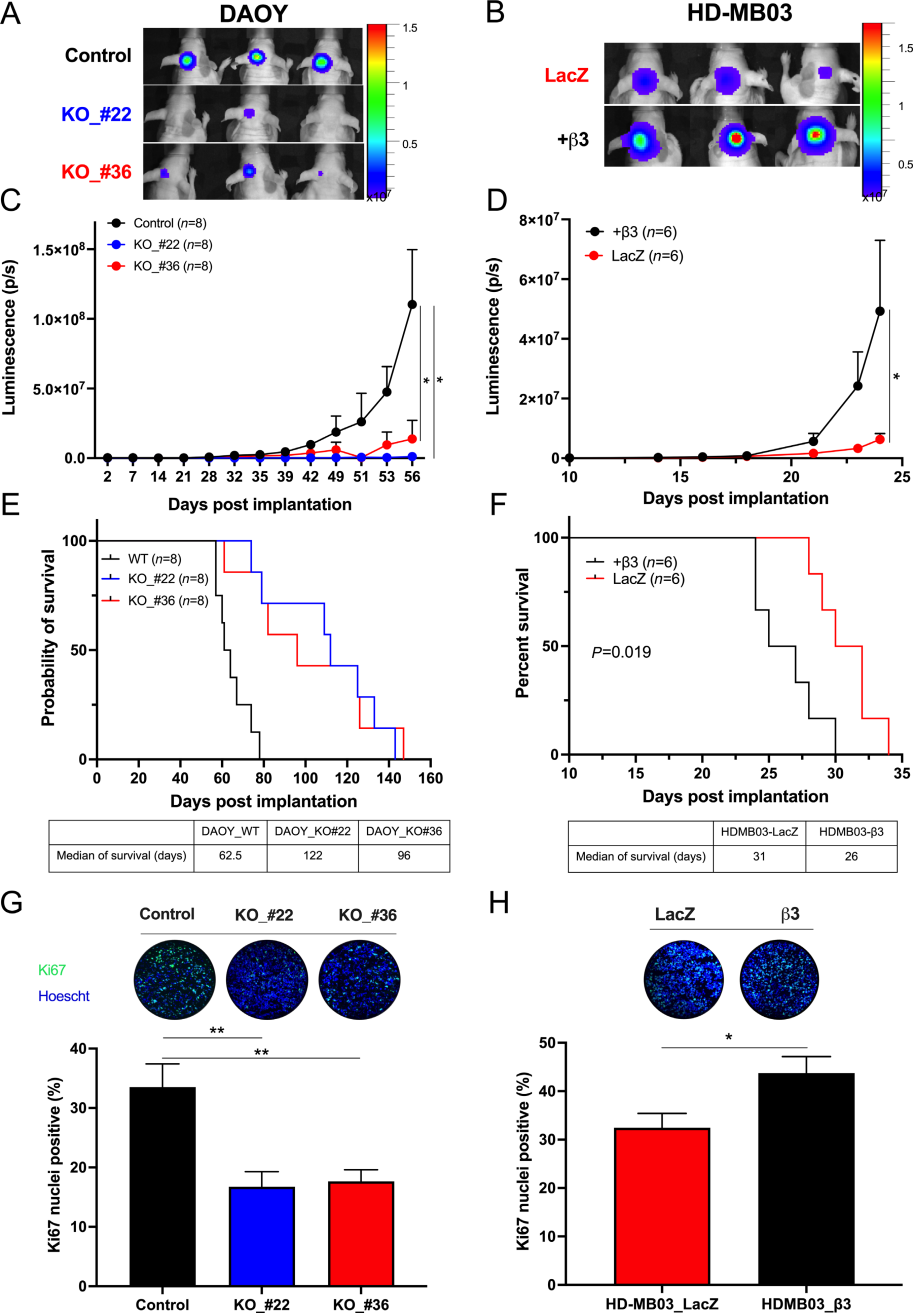
Integrin-αvβ3 promotes medulloblastoma intracranial tumor growth rate and increases survival. Representative images of BLI signal intensity 56 days (DAOY_control vs. KO_#22 and KO_#36; **A**) and 23 days (HD-MB03-LacZ vs. β3+; **B**) implantation. Three mice are shown per condition. Tumor growth of DAOY_control compared with KO_#22 and KO_#36 (**C**) and HD-MB03-LacZ compared with β3+ (**D**) measured by luciferase activity. Photon flux was quantified and analyzed using the IVIS imaging system. Survival curves of mice orthotopically implanted with DAOY_control vesrus KO_#22 and KO_#36 (**E**) and HD-MB03-LacZ versus β3+ (**F**) in the cerebellum. Day 0 corresponds to the day of tumor implantation. The median of survival is shown at the bottom of the graphs. The *P* value is indicated in the graph (log-rank test). Ki67 labeling of DAOY_control versus KO_#22 and KO_#36 (**G**) and HD-MB03-LacZ versus β3+ (**H**). Representative images of Ki67 immunostaining (green) and Hoechst33342 nuclear DNA counterstaining (blue) are shown at the top of the graphs. Key: *, *P* < 0.05; **, *P* < 0.01 versus control.

The fundamental role of integrin-αvβ3 in tumorigenesis features prompted us to investigate its role *in vivo* in orthotopic medulloblastoma models (Fig. 2). β3-depletion in DAOY cells impaired orthotopic tumor growth (Fig. 2A–C), increasing the median survival of mice bearing DAOY-KO_#22 and KO_#36 tumors by 95% and 54%, respectively, compared with control (*P* < 0.001 vs. DAOY_control; Fig. 2E). In HD-MB03 xenografts, β3 expression increased tumor growth rate and decreased mouse survival by 20% compared with control tumors (Fig. 2B, D, and F). Consistent with these observations, *ex vivo* analyses show an increase in Ki67-positive nuclei in β3-expressing DAOY or HD-MB03 tumors (Fig. 2G and H). Altogether, these results highlight the protumorigenic role of integrin-αvβ3 in medulloblastoma.

### Cilengitide and LM609 Disrupt αvβ3 Signaling in DAOY Cells and Recapitulates the β3-depletion Phenotype

**FIGURE 3:**
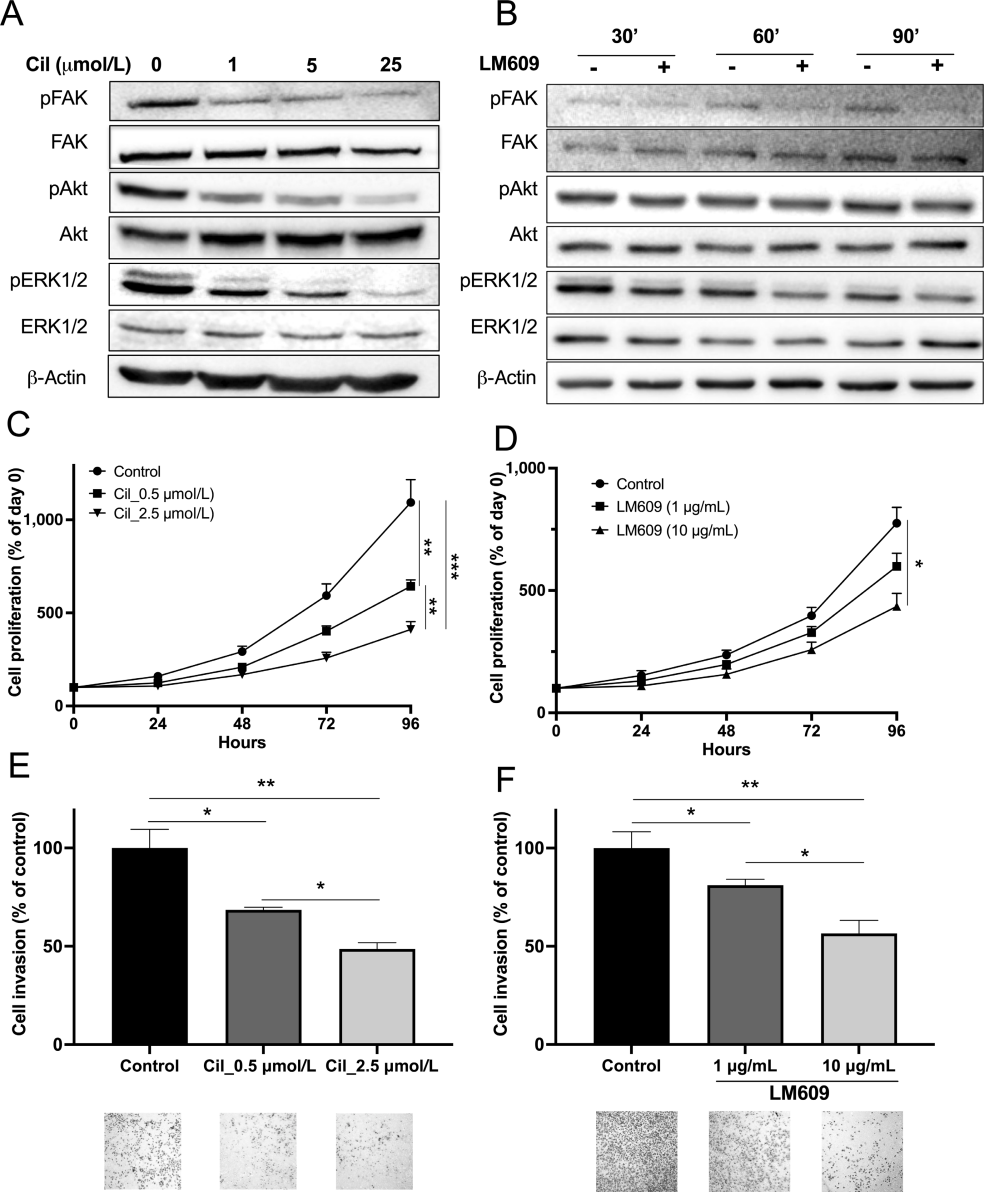
Pharmacologic disruption of integrin-αvβ3 impairs downstream signaling, decreasing cell proliferation, migration, and invasion of DAOY. Representative Western blots depicting FAK, Akt, and ERK1/2 activation in DAOY cells following treatment with cilengitide (1 hour of treatment with 1, 5, or 25 µmol/L cilengitide) in **A** or LM609 (30, 60, or 90 minutes of treatment with 10 µg/mL LM609) in **B**. Three independent experiments were conducted. Evaluation of DAOY cell proliferation after treatment with cilengitide (0.5 or 2.5 µmol/L) in **C** or LM609 (1 and 10 µg/mL) in **D** for 96 hours. Proliferation rates are expressed as a percentage of day 0. Three independent experiments were performed, and data are presented as mean ± SEM. Invasion assays for DAOY cells treated with cilengitide (**E**) or LM609 (**F**) for 12 hours. Serum-starved cells were exposed to cilengitide (0.5 or 2.5 µmol/L) in E or LM609 (1 and 10 µg/mL) in F and allowed to invade for 12 hours. Invasion assays were conducted using Matrigel-coated Boyden chamber assays, and the results are expressed as a percentage relative to control cells. Three independent experiments were carried out, and data are presented as mean ± SEM. Key: *, *P* < 0.05; **, *P* < 0.01; ***, *P* < 0.001 versus indicated conditions.

To mimic β3-depletion, we explored the pharmacologic impact of cilengitide, an RGD-derived compound, on medulloblastoma cell lines *in vitro* (Fig. 3). The IC_50_ of cilengitide was determined using MTT or adhesion assays. In both assays, the effect of cilengitide was restricted to β3-expressing medulloblastoma cell lines with IC_50_s of <2 and <10 µmol/L in DAOY-WT and HD-MB03-β3+, respectively (Supplementary Fig. S4). Consistent with the genetic approach, cilengitide impaired β3-downstream signaling in DAOY cells in a dose-dependent manner (Fig. 3A). This effect resulted in antiproliferative and anti-invasive effects at 0.5 and 2.5 µmol/L (Fig. 3C–E). Moreover, 48 hours treatment with cilengitide induced cell apoptosis in DAOY-WT cells (Supplementary Fig. S5).

To substantiate this observed phenotype, we conducted parallel investigations employing an anti-integrin-αvβ3 specific antibody (LM609 clone). LM609 treatment decreased impaired β3-downstream signaling in DAOY cells (Fig. 3B). This led to decrease in cell adhesion, invasion, and proliferation of DAOY_WT cells with no impact on DAOY_KO#22 (Fig. 3D and E, Supplementary Fig. S4–S6).

### *In Vivo* Antitumor Effects of Cilengitide are Restricted to αvβ3-expressing Medulloblastoma

**FIGURE 4:**
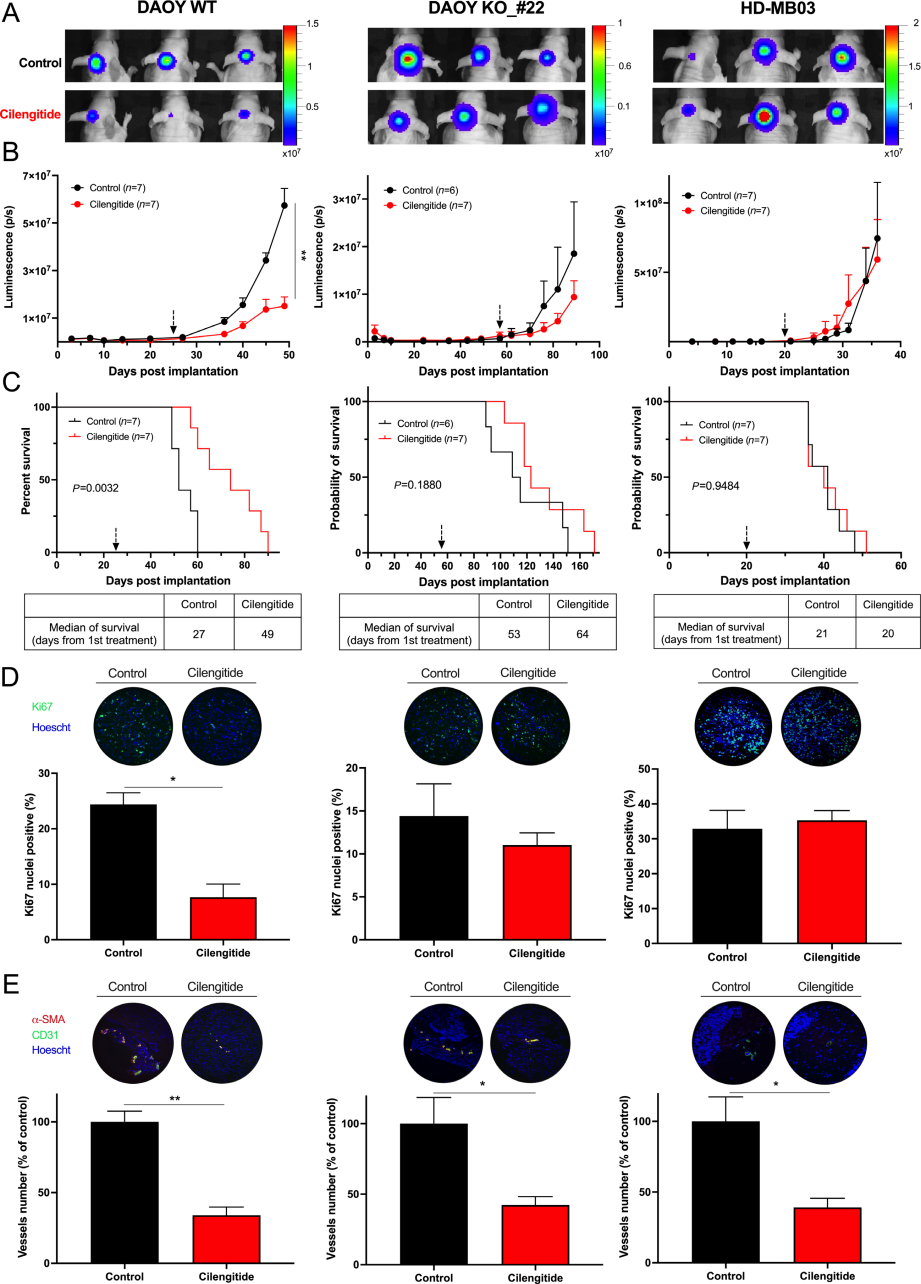
Antitumor effects of cilengitide are restricted to integrin-αvβ3–positive tumors. **A,** Representative images of the BLI signal intensity 49 days (DAOY_control, left), 89 days (DAOY-KO#22, middle), and 36 days (HD-MB03_WT, right) after implantation, corresponding to the time of first death. Three mice are shown per condition. **B,** Tumor growth of DAOY_control (left), DAOY-KO#22 (middle), and HD-MB03_WT (right) as assessed by luciferase activity. Photon flux was quantified and analyzed using the IVIS imaging system. **C,** Survival curves of mice orthotopically implanted with DAOY_control (left), DAOY KO_#22 (middle), and HD-MB03 (right) cells in the cerebellum and treated with cilengitide. Day 0 corresponds to tumor implantation, and the black arrow indicates the start of treatment. Mice were treated with 300 µg cilengitide three times a week. The median survival (from the start of treatment) is shown at the bottom of the graphs. The *P* value is indicated in the graph (log-rank test). **D,** Ki67 labeling of DAOY_control (left), DAOY-KO_#22 (center), and HD-MB03_WT (right) tumor sections. Representative images of Ki67 immunolabeling (green) and Hoechst33342 nuclear DNA counterstaining (blue) are shown at the top of the graphs. **E,** Vasculature labeling of DAOY_control (left), DAOY-KO_#22 (middle), and HD-MB03_WT (right) tumor sections. Vessels were identified by CD31 and αSMA immunofluorescent staining (green and magenta, respectively). The number of vessels (CD31^+^ and αSMA^+^) was determined, and results are expressed as the percentage of control conditions. Key: *, *P* < 0.05; **, *P* < 0.01 versus control.

Subsequently, we assessed the efficacy of cilengitide in orthotopic models of DAOY-WT, DAOY-KO, HD-MB03_WT, and HD-MB03_β3+ tumors to explore its relevance for integrin-αvβ3–expressing medulloblastomas (Fig. 4). Cilengitide treatment resulted in a delay in tumor growth only in mice with DAOY-WT or HD-MB03_β3+ tumors (Fig. 4A and B; Supplementary Fig. S7). This antitumor effect translated into a significant increase in mouse survival, with an 81% and 46% extension of survival observed in the DAOY-WT and HD-MB03_β3+ models, respectively (Fig. 4C, left; Supplementary Fig. S7C). Conversely, no significant effects were observed in the DAOY-KO and HD-MB03 tumors, underscoring the specificity of cilengitide for tumoral integrin-αvβ3 (Fig. 4A–C).

Because of its antiangiogenic function, cilengitide administration resulted in a 60% decrease in intratumoral blood vessels in each model (Fig. 4E). However, a decrease in Ki67 nuclei was observed only in DAOY-WT tumors, consistent with cilengitide's antitumor effect (Fig. 4D, left).

### SPECT-MRI as a Dual-modality Strategy for Measuring Integrin-αvβ3 Expression

**FIGURE 5:**
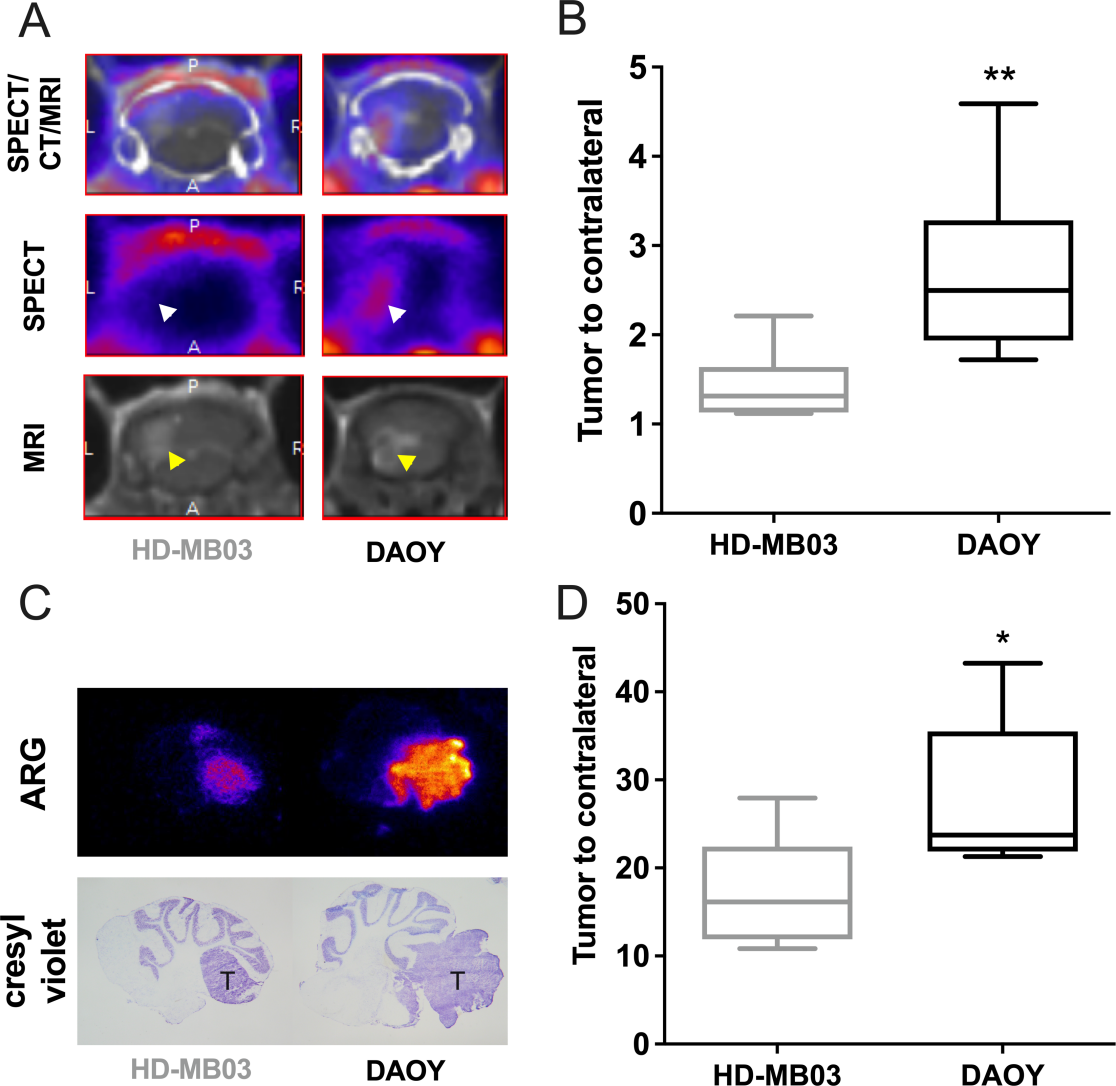
*In vivo* measurement of integrin-αvβ3 expression using SPECT/MRI dual-modality. **A,** Representative coronal views of MRI (bottom), SPECT (center), and fused SPECT/CT/MRI of HD-MB03 (left) and DAOY (right) tumors at 1 hour postinjection of ^99m^Tc-RAFT-RGD. **B,***In vivo* quantification of ^99m^Tc-RAFT-RGD tumor uptake from SPECT images. Results are expressed as tumor-to-cerebellum ratios. **C,** Autoradiography on 20 µm tumor-containing cerebellum slices (top) and staining of adjacent slices by Crystal Violet. **D,** Autoradiographic image quantification of ^99m^Tc-RAFT-RGD uptake in HD-MB03 and DAOY tumor lesions. Results are expressed as tumor-to-cerebellum ratios. Key: *, *P* < 0.05; **, *P* < 0.01 versus HD-MB03.

Cilengitide's restricted efficacy for αvβ3-positive tumors highlights the need for patient stratification. As a key component of personalized medicine, nuclear imaging is a relevant strategy to answer this issue. Therefore, we investigated the ability of ^99m^Tc-RAFT-RGD, a radioligand targeting integrin-αvβ3, to noninvasively evaluate its expression in DAOY and HD-MB03 orthotopic tumors (Fig. 5). The blood–brain barrier permeability was found to be permeable in both tumor models as demonstrated by gadolinium induced enhancement in T1-weighted fast spin-echo images. SPECT-MRI acquisitions showed that ^99m^Tc-RAFT-RGD uptake was readily observable in DAOY tumors, while no signal was found in HD-MB03 tumors (Fig. 5A). Tumor-to-cerebellum ratios were significantly higher in DAOY tumors than in HD-MB03 tumors (*P* < 0.010; Fig. 5B). Autoradiography of tumor slices was consistent with SPECT imaging quantification, with higher tumor-to-cerebellum ratios in DAOY tumors (Fig. 5C and D). These results suggest that radiotracers targeting αvβ3, such as ^99m^Tc-RAFT-RGD, could be used as companion markers to identify responders before cilengitide administration.

### Integrin-αvβ3 is a Relevant Target for Radioresistant Medulloblastoma

**FIGURE 6:**
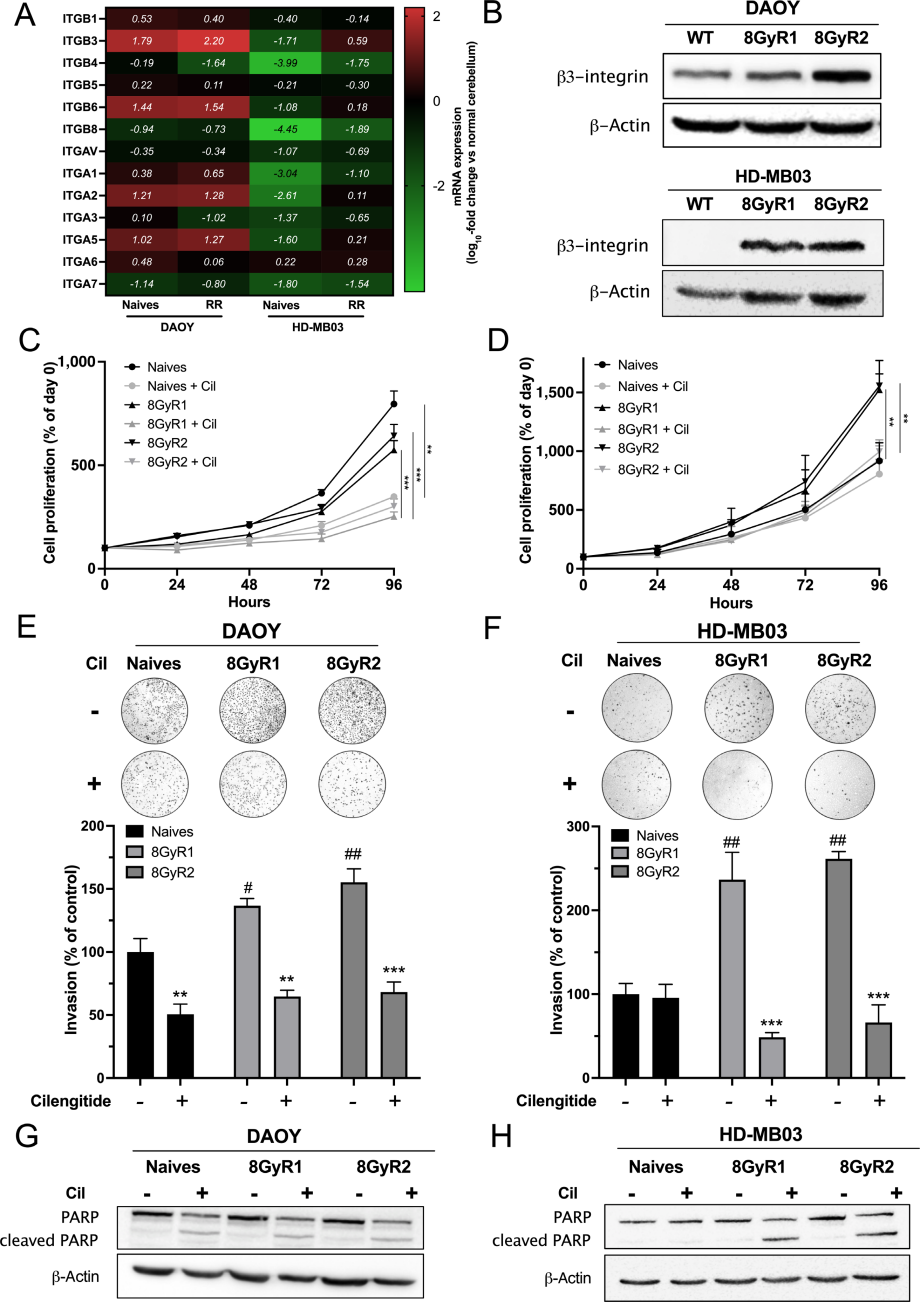
Radioresistant medulloblastomas overexpress integrin-αvβ3 and are sensitive to cilengitide. **A,** A heat map showing integrin gene expression in naïve versus radioresistant DAOY and HD-MB03 cells. Integrin expression was quantified by qRT-PCR. Results are expressed compared with normal cerebellum (log_10_-fold change). The means of three independent experiments are presented. **B,** Representative western blots of β3-integrin protein expression in naïve versus radioresistant DAOY (top) and HD-MB03 (bottom) cells. Three independent experiments were performed. The proliferation of naïve versus radioresistant DAOY (**C**) and HD-MB03 (**D**) cells treated with cilengitide (2.5 µmol/L) for 96 hours. Proliferation rates are expressed as the percentage of day 0. Three independent experiments were performed, and data are presented as mean ± SEM. Key: **, *P* < 0.01; ***, *P* < 0.001. Invasion assays of naïve versus radioresistant DAOY (**E**) and HD-MB03 (**F**) cells treated with cilengitide for 12 and 24 hours, respectively. Serum-starved cells were treated with cilengitide (2.5 µmol/L) and allowed to invade for 12 (DAOY) or 24 hours (HD-MB03). Invasions were conducted using Boyden chambers coated with Matrigel, and the results are expressed as the percentage of control naïve cells. Three independent experiments were performed, and data are presented as mean ± SEM. Key: **, *P* < 0.01; ***, *P* < 0.001 versus untreated cells; ^#^, *P* < 0.05; ^##^, *P* < 0.01 versus naïve cells. Effect of cilengitide on PARP cleavage. Naïve or radioresistant DAOY (**G**) and HD-MB03 (**H**) cells were treated with cilengitide (20 µmol/L) for 48 hours. Cell lysates were analyzed by Western blot analysis with an anti-PARP antibody. Three independent experiments were performed, with representative blots shown.

Because radioresistance is an important issue in brain tumor therapy, αvβ3-integrin expression was examined in radioresistant DAOY and HD-MB03 cells generated after multiple X-ray irradiations (Fig. 6). Cell survival was higher for radioresistant DAOY and HD-MB03 (DAOY-RR/HD-MB03-RR) than for naïve cells after a single irradiation (Supplementary Fig. S8). The mRNA analysis performed in these cells showed a significant increase in *ITGB3* expression in the radioresistant cells (Fig. 6A). This observation was confirmed at the protein level with higher expression in radioresistant cells (Fig. 6B).

The effect of cilengitide was further investigated *in vivo* in both models. It decreased cell proliferation of both naïve and radioresistant DAOY cells. However, its effect was limited to radioresistant HD-MB03 cells expressing integrin-αvβ3 (Fig. 6C and D). Invasion assays performed with these cells showed a significantly higher invasion potential for radioresistant cells than for naïve cells (Fig. 6E and F). Cilengitide decreased the number of invasive cells in the DAOY WT and radioresistant populations but only in the HD-MB03 radioresistant population. Cilengitide induced cell apoptosis in these cells, as shown by PARP cleavage after 24 hours of treatment (Fig. 6G and H). Altogether, these results suggest that αvβ3-integrin is a potential therapeutic target for radioresistant medulloblastoma.

## Discussion

Medulloblastoma stands as the most frequently diagnosed primary brain tumor among children (16, 17). The standard treatment protocol comprises maximal safe resection followed by craniospinal radiation and adjuvant therapy. However, approximately 30% of children with medulloblastoma experience disease relapse, which is almost always fatal (18). Therefore, recurrent medulloblastoma is a major therapeutic challenge. Given the apparent need to improve therapeutic strategies, considerable efforts have been made to understand the biology of brain tumors. The low survival rates of brain tumors, including medulloblastoma and GBM, are at least partly due to extensive brain tissue invasion.

Invasion is finely regulated and is mainly controlled by cancer and stromal cell interactions (19, 20). In addition to invasive features, GBM and medulloblastoma show marked tumor cell proliferation and increased angiogenesis. All these processes have mainly been studied in GBM, where integrins have been shown to be widely expressed and to play fundamental roles (21–23). Therefore, integrins have emerged as compelling targets for therapeutic intervention in GBM. Among them, the proangiogenic αvβ3 was the first to be found to be abundantly expressed in high-grade brain tumors (23).

Integrin-αvβ3 belongs to the integrin subtypes that recognize the tripeptide RGD sequence found in many ECM proteins, including fibronectin or vitronectin. Many studies have highlighted the role of αvβ3 in sustaining GBM's high proliferative, migrative, and invasive properties and promoting angiogenesis (24–26). Nevertheless, the expression and the role of integrin-αvβ3 in medulloblastoma remain relatively unexplored. In 2005, Lim and colleagues reported moderated integrin-αvβ3 expression in 5 patients with medulloblastoma (27). In our study, we detected a substantial integrin-αvβ3 expression in 20% of patient-derived samples, underscoring its significance for a subpopulation of patients with medulloblastoma. The restricted integrin-αvβ3 expression found in medulloblastoma-derived samples was corroborated in medulloblastoma cell lines, where only two of five exhibited *ITGB3* overexpression.

Within the SHH subgroups, DAOY and ONS-76 exhibited integrin expression, while D458 and HD-MB03 (group 3) as well as CHLA (group 4) did not display such expression. These findings imply the potential restriction of integrin-αvβ3 to a specific molecular subset of medulloblastomas. To validate this observation, further comprehensive studies are warranted. Afterward, the role of integrin-αvβ3 in medulloblastoma was explored through genetic manipulation: the β3-subunit was depleted in DAOY cells, and overexpression was induced via lentiviral transduction in DAOY-KO and HD-MB03 cells. While integrin-αvβ3 was conventionally associated with neovessels expression, IHC analysis of GBM samples revealed significant expression by tumor cells rather than angiogenic cells, thereby promoting GBM cell proliferation, migration, and invasion (23). In both cell lines, integrin-αvβ3 was a major player in these three processes. As orthotopic experiments showed, depleting the β3-subunit in two independent DAOY clones strongly reduced their tumorigenic potential. To a lesser extent, similar results were observed in HD-MB03 cells. These results support the original role of integrin-αvβ3 in medulloblastoma tumorigenesis. These results are in agreement with those obtained by Franovic and colleagues in orthotopic models of GBM using shRNA against β3-subunit (28).

Since its initial discovery, the core integrin-binding domain RGD in fibronectin has attracted considerable attention in the field of anticancer therapies. Among the various RGD-containing peptides developed to impair tumorigenesis, cilengitide was identified as a selective αvβ3 and αvβ5 inhibitor (29–31). This compound demonstrated antiangiogenic, cytotoxic, and anti-invasive activities in preclinical GBM models. Despite this promise, subsequent phase III CENTRIC and phase II CORE clinical trials showed no significant effects on OS in newly diagnosed GBM (12, 13). Nevertheless, in the CORE study, high αvβ3 levels in tumor cells were associated with improved OS. This finding suggests that cilengitide's efficiency was mediated by its action on αvβ3-positive tumor cells rather than angiogenic cells. Nonetheless, the mere presence of αvβ3-positive tumor cells might not prove adequate to render tumors receptive to integrin inhibition. For instance, recent findings by Cosset and colleagues*,* unveiled that the effectiveness of αvβ3 antagonists (such as cilengitide or LM609) in GBM models is confined to tumors reliant on GLUT3 (glucose transporter 3)-dependent glucose uptake (32).

In this study, we conducted an evaluation of cilengitide within our medulloblastoma models. The MTT assay showed that cilengitide did not affect HD-MB03 cells, while it strongly decreased the proliferation and viability of DAOY and HD-MB03-β3–overexpressing cells. The DAOY-KO cells were found to be only slightly affected by cilengitide treatment, with an IC_50_ >20-fold higher than that of DAOY-WT cells. This difference could be due to αvβ5 expression in DAOY-WT and -KO cells. Significantly, cilengitide exhibited a dose-dependent impairment of downstream integrin-αvβ3 signaling, evidenced by diminished phosphorylation of FAK, Akt, and ERK1/2. These molecular repercussions translated into noteworthy anti-proliferative, anti-migratory, and anti-invasive effects observed in DAOY.

Cilengitide was subsequently evaluated *in vivo* in orthotopic models of DAOY-WT, DAOY-KO, HD-MB03, and HD-MB03_β3+ models. As anticipated, cilengitide treatment led to a notable reduction in blood vessel density across our models. This aligns with cilengitide's well-documented potent antiangiogenic properties. However, the antitumor effect of cilengitide was observed exclusively in tumors with high integrin-αvβ3 expression (DAOY, and HD-MB03_β3+), leading to a significant increase in median survival in both models. This discovery underscores the critical importance of patient stratification before initiating cilengitide therapy, echoing findings from the CORE study conducted with patients with GBM. Further investigations are required to ascertain the mere presence of αvβ3 is adequate to confer tumor sensitivity to cilengitide. Given the intriguing observations made by Cosset and colleagues regarding the correlation between αvβ3-antagonist efficacy and GLUT3 addiction in GBM, it would be prudent to explore the transposition of these findings to the context of medulloblastoma. An option is to perform GLUT3 invalidation experiments to assess cilengitide effects in medulloblastoma models with suppressed GLUT3 expression. This could reveal whether the observed cilengitide sensitivity linked to αvβ3 presence is influenced by GLUT3 status in medulloblastoma.

Noninvasive molecular imaging presents a promising avenue for confirming target existence and monitoring tumor response through tracking its expression. Over recent decades, several αvβ3-targeting radiotracers have been developed and investigated for potential clinical application (8). Most are based on the tripeptide RGD due to its high affinity and specificity for integrin-αvβ3. Preclinical and clinical studies have found no physiologic brain uptake of different RGD peptides. However, clinical studies showed significant uptake in patients with medulloblastoma (8). In our study, we used a ^99m^Tc-radiolabeled tetrameric RGD-based peptide (RAFT-RGD) previously validated for imaging and internal vectorized therapy in preclinical GBM models (33). In alignment with our findings regarding cilengitide activity, our results revealed higher uptake of ^99m^Tc-RAFT-RGD in DAOY tumors compared with HD-MB03 tumors.

The combination of SPECT and MRI in these studies paves the way for nuclear imaging use in patients with medulloblastoma. Our results with radioresistant medulloblastoma suggest that integrin-αvβ3 is expressed in radioresistant tumors (34). Therefore, SPECT/MRI in recurrent medulloblastoma could be considered to determine eligibility for cilengitide treatment. Indeed, accumulating evidence supports the role of integrin-αvβ3 in resistance to conventional therapies. Other studies have reported integrin-αvβ3 expression in radioresistant gliomas and prostate cancer tumors (34, 35). If integrin-αvβ3 expression is an important determinant of cilengitide efficacy, several factors, such as redundancy or compensatory mechanisms, could explain its limited efficacy in the clinic.

The limited efficacy of cilengitide in clinical application suggests the significance of integrating integrin-αvβ3 imaging as a prerequisite before initiating treatment. Furthermore, it emphasizes the need to explore alternative strategies beyond pharmacologic inhibition. The remarkable advances in nuclear medicine, especially targeted therapies, may provide new tools for treating recurrent medulloblastoma. Most therapeutic radiopharmaceuticals are labeled with β-emitting isotopes. These particles penetrate only a few millimeters into tissue and allow irradiation of cells within a limited radius, resulting in tumor cell death while sparing surrounding healthy tissue. Commonly used β-emitters include lutetium-177 (^177^Lu), which has a half-life of 6.7 days and relatively high β-emission (0.497 MeV). These characteristics offer the advantage of delivering a high dose and achieving prolonged tumor irradiation. In addition, a unique feature of radionuclides is that they can exert a “cross-fire” effect that destroys neighboring cells. Several ^177^Lu-radiolabeled RGDs have been studied in preclinical GBM models. One, the ^177^Lu-NOTA-EB-RGD, was found to completely eradicate tumor growth from αvβ3-expressing patient xenografts (36). Although all these compounds require further clinical investigation, they may have great potential for integration into combined therapies for brain tumors, including medulloblastomas. Future perspectives of our study include the evaluation of ^177^Lu-RAFT-RGD in mouse medulloblastoma models, which could be the beginning of the era of theranostics in these tumors.

## Supplementary Material

Table S1 List of the primers used for qPCR analysis in this study.

Figure S1 Measurement of integrin-αvβ3 expression in MDB-derived cell lines performed by FACS. DAOY- and HD-MB03-derived cells were seeded in 6-well dishes for 24h. After detachment with accutase, cells were collected and incubated with an anti-αvβ3 antibody (ab190147, Abcam®) followed by a goat anti-mouse secondary antibody coupled to AlexaFluorTM488. Data were analyzed per sample using a BD FACSMelodyTM cytometer. Data were analyzed using FlowJoTM software.

Figure S2 ECM-mediated cell adhesion assay using crystal violet staining. DAOY-derived (A-B) and HD-MB03-derived (C) cells were allowed to attach to different ECM proteins coated on 48-wells plates for 30 min. Adherent cells were stained with crystal violet, solubilized with DMSO and quantified at OD560 nm. ** p<0.01 vs DAOY_Ctl or HD-MB03_LacZ; # p<0.05, ## p<0.01 vs DAOY_ β3-overexpressing cells.

Figure S3 Western-blot analysis of integrin-αvβ3-downstream pathways. Relative protein contents of pFAK/FAK, pAkt/Akt and pERK1/2/ERK were determined in DAOY-derived and HD-MB03-derived cells. Actin acted as a protein-loading control and blots are representative of three independent experiments.

Figure S4 Pharmacological disruption of integrin-αvβ3 decreased cell adhesion of integrin-αvβ3 positive MB cell lines. (A) The IC50s for cilengitide determined by MTT and adhesion assays. In the MTT assay, IC50s (μM) were determined after 48 h of exposure to cilengitide. In adhesion assays, 96-well plates were coated with fibronectin (1 μg/well), and cells were allowed to adhere in the presence of cilengitide for 2 h. The IC50s were determined after staining the cells with Crystal Violet (1%), resuspending them in DMSO, and absorbance measurement at 590 nm. (B) 96-well plates were coated with fibronectin (1μg/well), and cells were allowed to adhere in the presence of LM609 at the indicated concentrations for 2 h. Cells were stained the cells with Crystal Violet (1%), resuspending them in DMSO, and absorbance measurement at 590 nm. Key: *, p < 0.05; **, p < 0.01, *** p<0.001 vs control conditions.

Figure S5 Cilengitide led to DAOY apoptotic cell death. (A)Cell death was determined by the PI staining method. DAOY cells were treated with cilengitide (20 μM) for 48 h, then non-adherent and adherent cells were collected, and cell viability was assessed. Three independent experiments were performed, and data are presented as mean ± SEM. (B) The effect of cilengitide on PARP cleavage. DAOY cells were treated with cilengitide (20 μM) for 48 h. Cell lysates were analyzed by western blot with an anti-PARP antibody. Three independent experiments were performed, with representative blots shown. Key: *, p < 0.05; **, p < 0.01. vs control or indicated conditions; #, p < 0.05 vs Cil_1 μM.

Figure S6 Proliferation of DAOY_ KO#22 cells treated with LM609 (1 or 10 μg/mL) for 96 h. The proliferation rates are expressed as the percentage of day 0.

Figure S7 Cilengitide delays tumor growth in HD-MB03-β3+ orthotopic xenografts. A) Representative images of the BLI signal intensity 30 days after HD-MB03-β3+ tumor implantation, corresponding to the time of first death. Three mice are shown per condition. (B) Tumor growth of HD-MB03-β3+ assessed by luciferase activity. Photon flux was quantified and analyzed using the IVIS imaging system. (C) Survival curves of mice orthotopically implanted with HD-MB03-β3+ cells in the cerebellum and treated with cilengitide. Day 0 corresponds to tumor implantation, and the black arrow indicates the start of treatment. Mice were treated with 300 μg cilengitide three times a week. The median survival (from the start of treatment) is shown at the bottom of the graphs. The p-value is indicated in the graph (Log-rank test). Key: ** p < 0.01 vs. control

Figure S8 Colony formation assay performed with naïves and radioresistant cells. Naives and radioresistant DAOY (A) and HD-MB03 (B) cells were seeded after radiation treatment (0 to 8 Gy) into 60mm plates (2,000 and 4,000 cells respectively for DAOY and HD-MB03-derived populations). Once colonies visible (approximately 10 days post-seeding), cells were washed in PBS and colored with Giemsa for 30min. Plates were then washed and allowed to air dry before colonies were counted. Analysis was performed by calculating survival fractions.
