# Supplementary material for: Integrin-αvβ3 is a Therapeutically Targetable Fundamental Factor in Medulloblastoma Tumorigenicity and Radioresistance
Source: Cancer Res Commun. 2023 Dec 7;3(12):2483–96. doi: 10.1158/2767-9764.CRC-23-0298 (PMC10702273; doi:10.1158/2767-9764.CRC-23-0298)
Supplement: Table S1 — List of the primers used for qPCR analysis in this study. [file crc-23-0298-s01.pdf]

Oligo Name	Sequence (5'->3')
qPCR_ITGB1_For	GTAACCAACCGTAGCAAAGG
qPCR_ITGB1_Rev	CCCCTGATCTTAATCGCAAAAC
qPCR_ITGB3_For	GTAACCTGCGGATTGGCTTC
qPCR_ITGB3_Rev	GTCACCTGGTCAGTTAGCG
qPCR_ITGB4_For	GCAGCTTCCAAATCACAGAGG
qPCR_ITGB4_Rev	CCAGATCATCGGACATGGAG
qPCR_ITGB5_For	GGAAGTTCGGAAACAGAGGG
qPCR_ITGB5_Rev	CTTTCGCCAGCCAATCTTCTC
qPCR_ITGB6_For	CTCAACACAATAAAGGAGCTGG
qPCR_ITGB6_Rev	GGGGATACAGGTTTTTCCAC
qPCR_ITGB8_For	GTGAAAGTCATATCGGATGGCG
qPCR_ITGB8_Rev	GCTATCAAGAGCGAGATGAGAC
qPCR_ITGAV_For	GCTGTCGGAGATTTCAATGG
qPCR_ITGAV_Rev	CTGCTCGCCAGTAAAATTG
qPCR_ITGA1_For	CTGGACATAGTCATAGTGCTGG
qPCR_ITGA1_Rev	ACCTGTGTCTGTTTAGGACC
qPCR_ITGA2_For	GGGAATCAGTATTACACAACGGG
qPCR_ITGA2_Rev	CACAACATCTATGAGGGAAGGG
qPCR_ITGA3_For	CAACCTGGATACCCGATTCC
qPCR_ITGA3_Rev	GCTCTGTCTGCCGATGGAG
qPCR_ITGA5_For	GGCTTCAACTTAGACGCGGAG
qPCR_ITGA5_Rev	TGGCTGGTATTAGCCTTGGGT
qPCR_ITGA6_For	CAGTGGAGCCGTGGTTTTG
qPCR_ITGA6_Rev	CCACCGCCACATCATAGCC
qPCR_ITGA7_For	CTGACTCCATGTTCGGGATC
qPCR_ITGA7_Rev	CACCTGTGAAGGTTTGGCG
qPCR_h36B4_For	GCAGATTGGCTACCCAACTG
qPCR_h36B4_Rev	GGAAGGTGTAATCCGTCTCC

Table S1. List of the primers used for qPCR analysis in this study.
