# Supplementary material for: Integrin-αvβ3 is a Therapeutically Targetable Fundamental Factor in Medulloblastoma Tumorigenicity and Radioresistance
Source: Cancer Res Commun. 2023 Dec 7;3(12):2483–96. doi: 10.1158/2767-9764.CRC-23-0298 (PMC10702273; doi:10.1158/2767-9764.CRC-23-0298)
Supplement: Figure S1 — Measurement of integrin-αvβ3 expression in MDB-derived cell lines performed by FACS. DAOY- and HD-MB03-derived cells were seeded in 6-well dishes for 24h. After detachment with accutase, cells were collected and incubated with an anti-αvβ3 antibody (ab190147, Abcam®) followed by a goat anti-mouse secondary antibody coupled to AlexaFluorTM488. Data were analyzed per sample using a BD FACSMelodyTM cytometer. Data were analyzed using FlowJoTM software. [file crc-23-0298-s02.pdf]

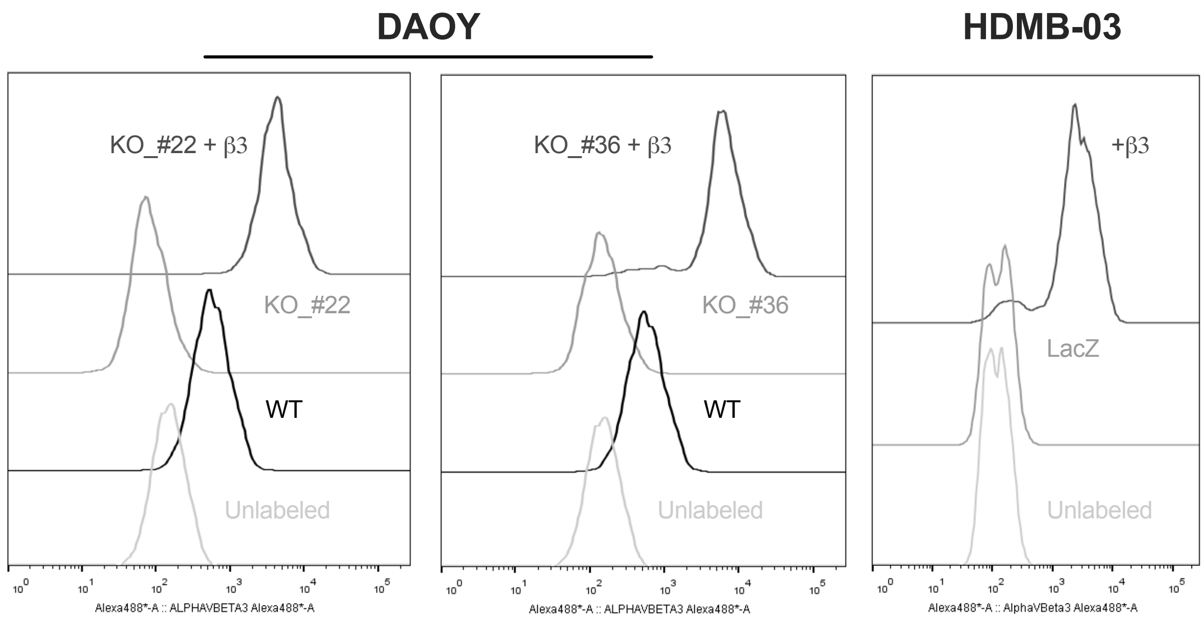

Figure S1. Measurement of integrin-αvβ3 expression in MDB-derived cell lines performed by FACS. DAOY- and HD-MB03-derived cells were seeded in 6-well dishes for 24h. After detachment with accutase, cells were collected and incubated with an anti-αvβ3 antibody (ab190147, Abcam®) followed by a goat anti-mouse secondary antibody coupled to AlexaFluor™488. Data were analyzed per sample using a BD FACSMelody™ cytometer. Data were analyzed using FlowJo™ software.
