# Supplementary material for: Integrin-αvβ3 is a Therapeutically Targetable Fundamental Factor in Medulloblastoma Tumorigenicity and Radioresistance
Source: Cancer Res Commun. 2023 Dec 7;3(12):2483–96. doi: 10.1158/2767-9764.CRC-23-0298 (PMC10702273; doi:10.1158/2767-9764.CRC-23-0298)
Supplement: Figure S2 — ECM-mediated cell adhesion assay using crystal violet staining. DAOY-derived (A-B) and HD-MB03-derived (C) cells were allowed to attach to different ECM proteins coated on 48-wells plates for 30 min. Adherent cells were stained with crystal violet, solubilized with DMSO and quantified at OD560 nm. ** p<0.01 vs DAOY_Ctl or HD-MB03_LacZ; # p<0.05, ## p<0.01 vs DAOY_ β3-overexpressing cells. [file crc-23-0298-s03.pdf]

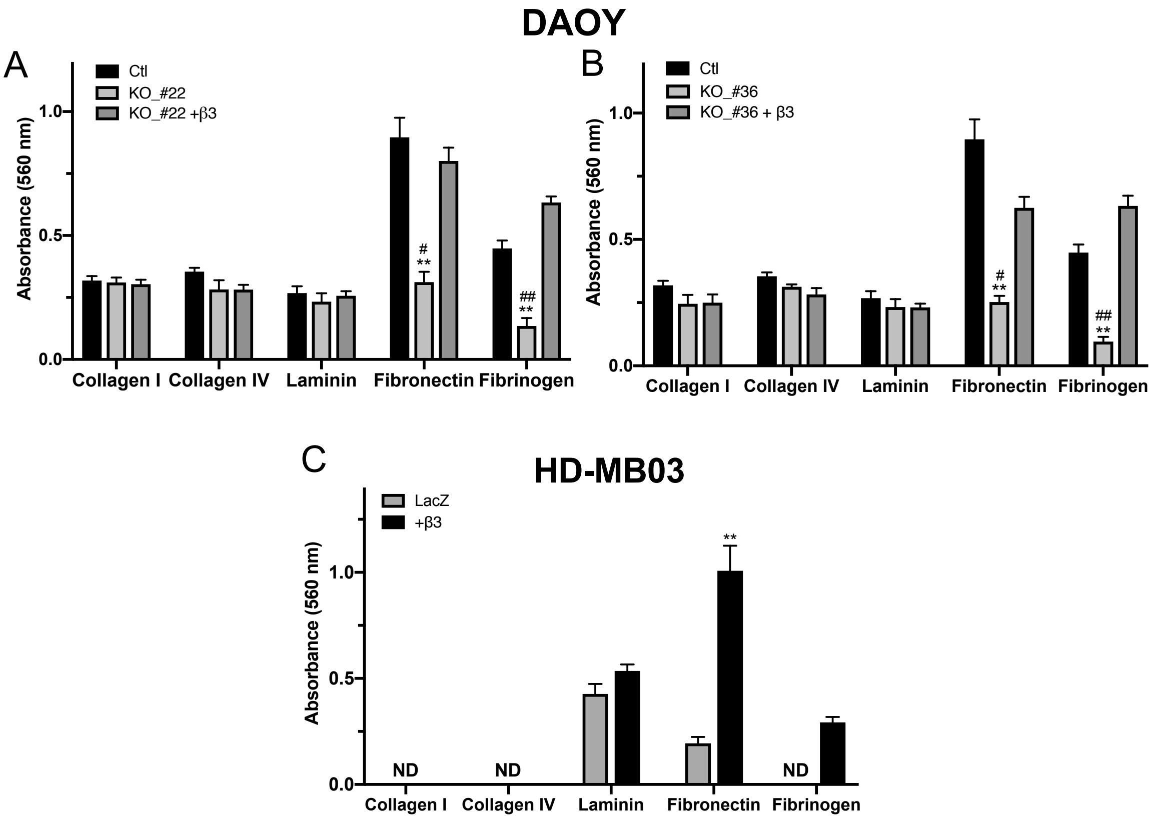

Figure S2. ECM-mediated cell adhesion assay using crystal violet staining. DAOY-derived (**A-B**) and HD-MB03-derived (**C**) cells were allowed to attach to different ECM proteins coated on 48-wells plates for 30 min. Adherent cells were stained with crystal violet, solubilized with DMSO and quantified at OD560 nm. ** $p<0.01$ vs DAOY_Ctl or HD-MB03_LacZ; # $p<0.05$, ## $p<0.01$ vs DAOY_ β3-overexpressing cells.
