# Supplementary material for: Integrin-αvβ3 is a Therapeutically Targetable Fundamental Factor in Medulloblastoma Tumorigenicity and Radioresistance
Source: Cancer Res Commun. 2023 Dec 7;3(12):2483–96. doi: 10.1158/2767-9764.CRC-23-0298 (PMC10702273; doi:10.1158/2767-9764.CRC-23-0298)
Supplement: Figure S3 — Western-blot analysis of integrin-αvβ3-downstream pathways. Relative protein contents of pFAK/FAK, pAkt/Akt and pERK1/2/ERK were determined in DAOY-derived and HD-MB03-derived cells. Actin acted as a protein-loading control and blots are representative of three independent experiments. [file crc-23-0298-s04.pdf]

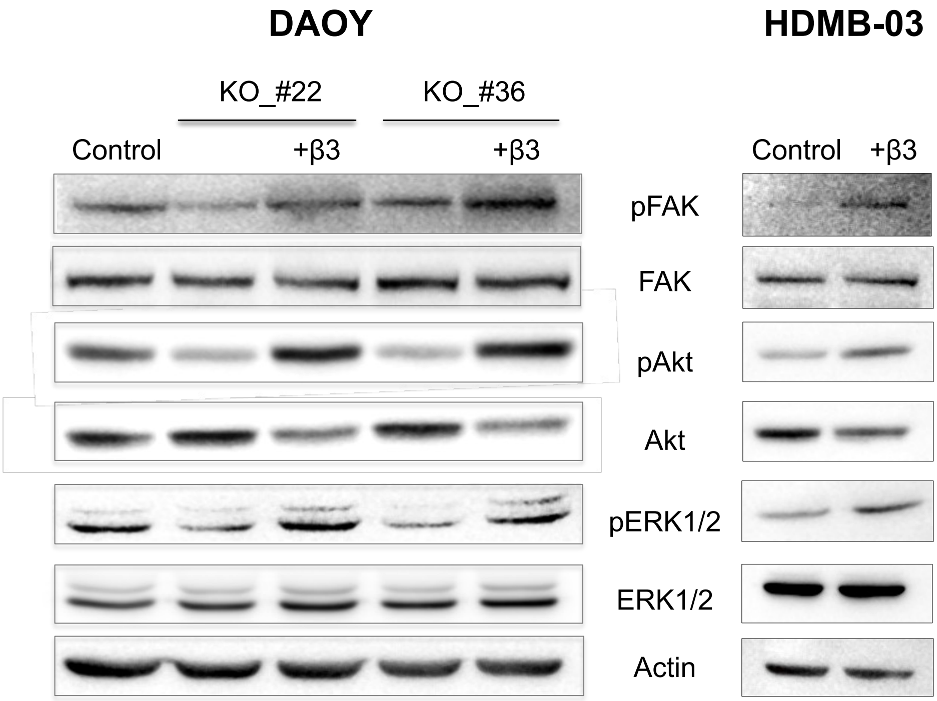

Figure S3. Western-blot analysis of integrin-αvβ3-downstream pathways. Relative protein contents of pFAK/FAK, pAkt/Akt and pERK1/2/ERK were determined in DAOY-derived and HD-MB03-derived cells. Actin acted as a protein-loading control and blots are representative of three independent experiments.
