# Supplementary material for: Integrin-αvβ3 is a Therapeutically Targetable Fundamental Factor in Medulloblastoma Tumorigenicity and Radioresistance
Source: Cancer Res Commun. 2023 Dec 7;3(12):2483–96. doi: 10.1158/2767-9764.CRC-23-0298 (PMC10702273; doi:10.1158/2767-9764.CRC-23-0298)
Supplement: Figure S4 — Pharmacological disruption of integrin-αvβ3 decreased cell adhesion of integrin-αvβ3 positive MB cell lines. (A) The IC50s for cilengitide determined by MTT and adhesion assays. In the MTT assay, IC50s (μM) were determined after 48 h of exposure to cilengitide. In adhesion assays, 96-well plates were coated with fibronectin (1 μg/well), and cells were allowed to adhere in the presence of cilengitide for 2 h. The IC50s were determined after staining the cells with Crystal Violet (1%), resuspending them in DMSO, and absorbance measurement at 590 nm. (B) 96-well plates were coated with fibronectin (1μg/well), and cells were allowed to adhere in the presence of LM609 at the indicated concentrations for 2 h. Cells were stained the cells with Crystal Violet (1%), resuspending them in DMSO, and absorbance measurement at 590 nm. Key: *, p < 0.05; **, p < 0.01, *** p<0.001 vs control conditions. [file crc-23-0298-s05.pdf]

A

	Assay	DAOY_WT	DAOY_KO#22	HD-MB03_LacZ	HD-MB03_β3
**IC50 (µM)**	MTT	1.9 ± 0.4	45.8 ± 8.2	> 200	9.2 ± 1.8
	Adhesion	0.8 ± 0.1	18.7 ± 5.3	46.9 ± 7.4	5.2 ± 1.3

B

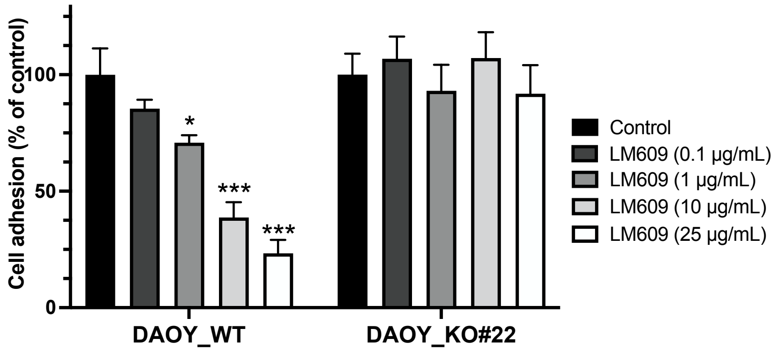

Figure S4. Pharmacological disruption of integrin-αvβ3 decreased cell adhesion of integrin-αvβ3 positive MB cell lines. **(A)** The IC50s for cilengitide determined by MTT and adhesion assays. In the MTT assay, IC50s (µM) were determined after 48 h of exposure to cilengitide. In adhesion assays, 96-well plates were coated with fibronectin (1 µg/well), and cells were allowed to adhere in the presence of cilengitide for 2 h. The IC50s were determined after staining the cells with Crystal Violet (1%), resuspending them in DMSO, and absorbance measurement at 590 nm. **(B)** 96-well plates were coated with fibronectin (1µg/well), and cells were allowed to adhere in the presence of LM609 at the indicated concentrations for 2 h. Cells were stained the cells with Crystal Violet (1%), resuspending them in DMSO, and absorbance measurement at 590 nm. Key: *, $p < 0.05$; **, $p < 0.01$, *** $p<0.001$ vs control conditions.
