# Supplementary material for: Integrin-αvβ3 is a Therapeutically Targetable Fundamental Factor in Medulloblastoma Tumorigenicity and Radioresistance
Source: Cancer Res Commun. 2023 Dec 7;3(12):2483–96. doi: 10.1158/2767-9764.CRC-23-0298 (PMC10702273; doi:10.1158/2767-9764.CRC-23-0298)
Supplement: Figure S5 — Cilengitide led to DAOY apoptotic cell death. (A)Cell death was determined by the PI staining method. DAOY cells were treated with cilengitide (20 μM) for 48 h, then non-adherent and adherent cells were collected, and cell viability was assessed. Three independent experiments were performed, and data are presented as mean ± SEM. (B) The effect of cilengitide on PARP cleavage. DAOY cells were treated with cilengitide (20 μM) for 48 h. Cell lysates were analyzed by western blot with an anti-PARP antibody. Three independent experiments were performed, with representative blots shown. Key: *, p < 0.05; **, p < 0.01. vs control or indicated conditions; #, p < 0.05 vs Cil_1 μM. [file crc-23-0298-s06.pdf]

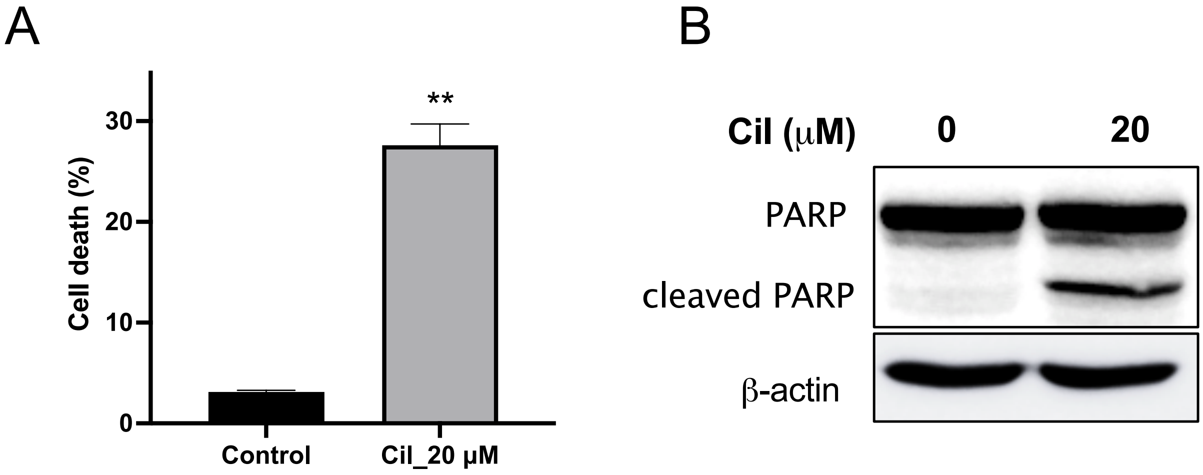

Figure S5. Cilengitide led to DAOY apoptotic cell death. (A) Cell death was determined by the PI staining method. DAOY cells were treated with cilengitide (20 μM) for 48 h, then non-adherent and adherent cells were collected, and cell viability was assessed. Three independent experiments were performed, and data are presented as mean ± SEM. **(B)** The effect of cilengitide on PARP cleavage. DAOY cells were treated with cilengitide (20 μM) for 48 h. Cell lysates were analyzed by western blot with an anti-PARP antibody. Three independent experiments were performed, with representative blots shown. Key: *, $p < 0.05$; **, $p < 0.01$. vs control or indicated conditions; #, $p < 0.05$ vs Cil_1 μM.
