# Supplementary material for: Integrin-αvβ3 is a Therapeutically Targetable Fundamental Factor in Medulloblastoma Tumorigenicity and Radioresistance
Source: Cancer Res Commun. 2023 Dec 7;3(12):2483–96. doi: 10.1158/2767-9764.CRC-23-0298 (PMC10702273; doi:10.1158/2767-9764.CRC-23-0298)
Supplement: Figure S6 — Proliferation of DAOY_ KO#22 cells treated with LM609 (1 or 10 μg/mL) for 96 h. The proliferation rates are expressed as the percentage of day 0. [file crc-23-0298-s07.pdf]

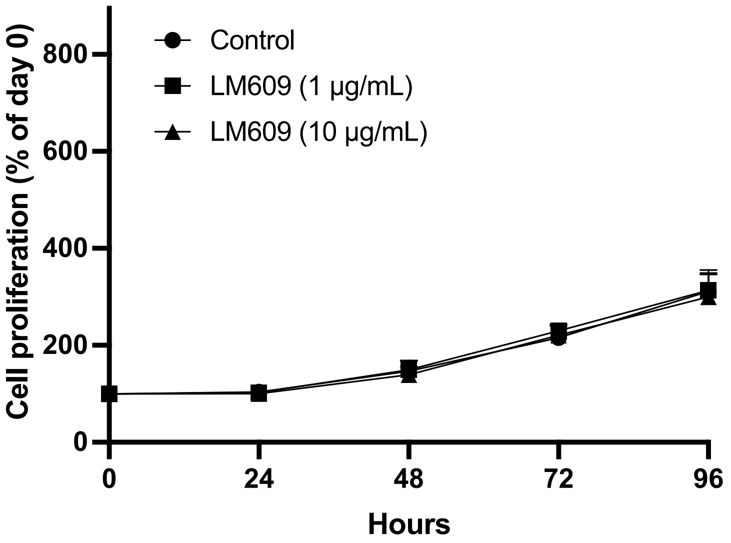

Figure S6. Proliferation of DAOY_ KO#22 cells treated with LM609 (1 or 10 µg/mL) for 96 h. The proliferation rates are expressed as the percentage of day 0.
