# Supplementary material for: Integrin-αvβ3 is a Therapeutically Targetable Fundamental Factor in Medulloblastoma Tumorigenicity and Radioresistance
Source: Cancer Res Commun. 2023 Dec 7;3(12):2483–96. doi: 10.1158/2767-9764.CRC-23-0298 (PMC10702273; doi:10.1158/2767-9764.CRC-23-0298)
Supplement: Figure S7 — Cilengitide delays tumor growth in HD-MB03-β3+ orthotopic xenografts. A) Representative images of the BLI signal intensity 30 days after HD-MB03-β3+ tumor implantation, corresponding to the time of first death. Three mice are shown per condition. (B) Tumor growth of HD-MB03-β3+ assessed by luciferase activity. Photon flux was quantified and analyzed using the IVIS imaging system. (C) Survival curves of mice orthotopically implanted with HD-MB03-β3+ cells in the cerebellum and treated with cilengitide. Day 0 corresponds to tumor implantation, and the black arrow indicates the start of treatment. Mice were treated with 300 μg cilengitide three times a week. The median survival (from the start of treatment) is shown at the bottom of the graphs. The p-value is indicated in the graph (Log-rank test). Key: ** p < 0.01 vs. control [file crc-23-0298-s08.pdf]

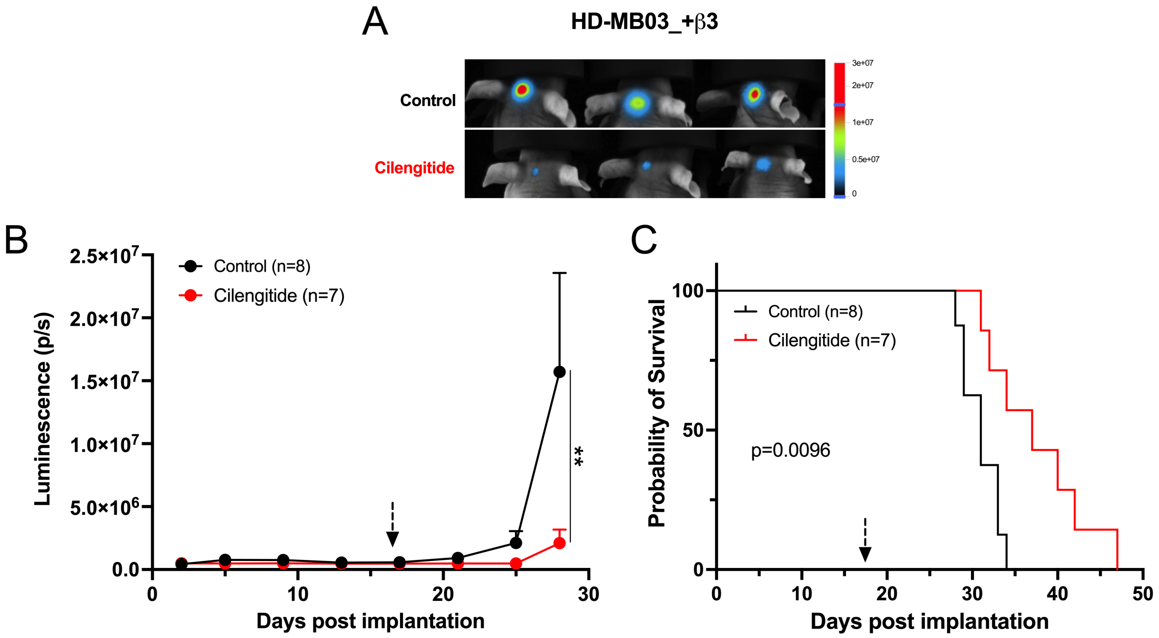

	Control	Cilengitide
Median of survival (days from 1st treatment)	13	19

Figure S7. Cilengitide delays tumor growth in HD-MB03-β3+ orthotopic xenografts. A) Representative images of the BLI signal intensity 30 days after HD-MB03-β3+ tumor implantation, corresponding to the time of first death. Three mice are shown per condition. (B) Tumor growth of HD-MB03-β3+ assessed by luciferase activity. Photon flux was quantified and analyzed using the IVIS imaging system. (C) Survival curves of mice orthotopically implanted with HD-MB03-β3+ cells in the cerebellum and treated with cilengitide. Day 0 corresponds to tumor implantation, and the black arrow indicates the start of treatment. Mice were treated with 300 µg cilengitide three times a week. The median survival (from the start of treatment) is shown at the bottom of the graphs. The p-value is indicated in the graph (Log-rank test). Key: ** $p < 0.01$ vs. control
