# Supplementary material for: Integrin-αvβ3 is a Therapeutically Targetable Fundamental Factor in Medulloblastoma Tumorigenicity and Radioresistance
Source: Cancer Res Commun. 2023 Dec 7;3(12):2483–96. doi: 10.1158/2767-9764.CRC-23-0298 (PMC10702273; doi:10.1158/2767-9764.CRC-23-0298)
Supplement: Figure S8 — Colony formation assay performed with naïves and radioresistant cells. Naives and radioresistant DAOY (A) and HD-MB03 (B) cells were seeded after radiation treatment (0 to 8 Gy) into 60mm plates (2,000 and 4,000 cells respectively for DAOY and HD-MB03-derived populations). Once colonies visible (approximately 10 days post-seeding), cells were washed in PBS and colored with Giemsa for 30min. Plates were then washed and allowed to air dry before colonies were counted. Analysis was performed by calculating survival fractions. [file crc-23-0298-s09.pdf]

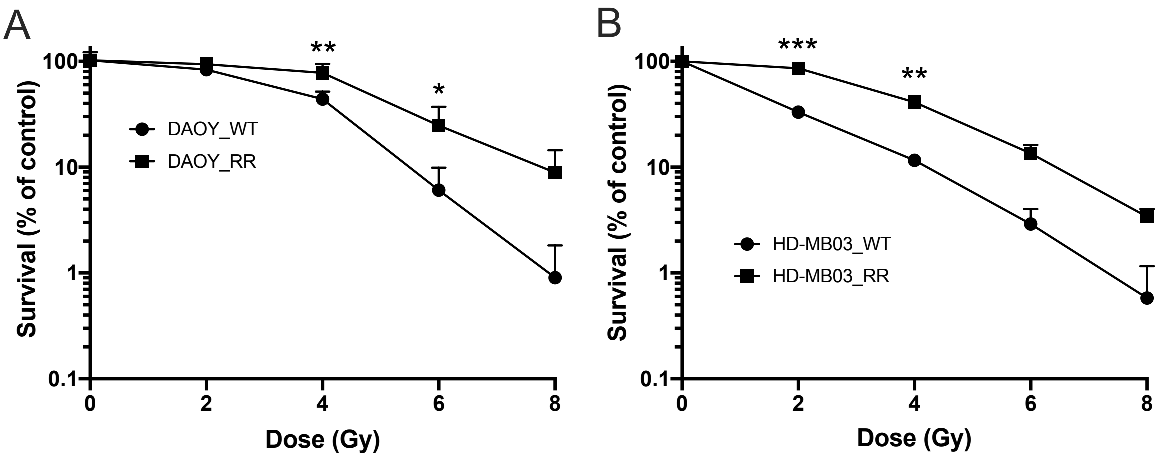

Figure S8. Colony formation assay performed with naïves and radioresistant cells. Naives and radioresistant DAOY (A) and HD-MB03 (B) cells were seeded after radiation treatment (0 to 8 Gy) into 60mm plates (2,000 and 4,000 cells respectively for DAOY and HD-MB03-derived populations). Once colonies visible (approximately 10 days post-seeding), cells were washed in PBS and colored with Giemsa for 30min. Plates were then washed and allowed to air dry before colonies were counted. Analysis was performed by calculating survival fractions.
